# Structure and trends of international sport nutrition research between 2000 and 2018: bibliometric mapping of sport nutrition science

**DOI:** 10.1186/s12970-021-00409-5

**Published:** 2021-02-05

**Authors:** Anna Kiss, Ágoston Temesi, Orsolya Tompa, Zoltán Lakner, Sándor Soós

**Affiliations:** 1 Department of Science Policy and Scientometrics, Library and Information Centre of the Hungarian Academy of Sciences (MTA), Arany János street 1, Budapest, 1050 Hungary; 2 Faculty of Education and Psychology, Eötvös Loránd University, Budapest, Hungary; 3 Institute of Agribusiness, Department of Food Chain Management, Faculty of Economics and Social Sciences, Szent István University, Gödöllő, Hungary

**Keywords:** Network text analysis, Scientometric analysis, Sport nutrition research trends, Bibliometric mapping

## Abstract

**Background:**

The tool kits of bibliometrics and science mapping provide a standard methodology to map the knowledge base of specific fields of science. The aim of the present research is the analysis of the recent international trends of sport nutrition science, as well as the primary identification of the research topics and results of sport nutrition science via enhanced bibliometric methods for the 2000–2018 time period.

**Methods:**

Altogether, 3889 publications were included in this study. We identified the most relevant sport nutrition topics by running a community detection algorithm on the proximity network constructed via network text analysis. The key issues and key concepts of sport nutrition topics as well as their relations were evaluated via network analysis. Besides, we carried a chronological analysis of topics out and a scientometric evaluative analysis was also created.

**Results:**

We identified the four main basic groups from which the 18 most characteristics topics were analyzed. The 18 topics are the following: ‘soccer and physiology’, ‘carbohydrate metabolism’, ‘muscle physiology: alkalosis and acidosis’, ‘muscle mass gain and dietary supplementation’, ‘fluid balance and hydration’, ‘dietary intake and nutrition knowledge’, ‘determination of energy need of athletes’, ‘bone health and female athlete triad’, ‘hydration strategy’, ‘body weight management’, ‘nutritional strategies and human skeletal muscle’, ‘dietary supplementation of nitrates’, ‘oxidative stress and dietary supplement use’, ‘dietary supplement use and doping’, ‘oxidative stress and inflammation and dietary antioxidants’, ‘exercise adaptation and nutritional strategies’, ‘gut microbiota’, ‘celiac disease’. Regarding the size of the topic, researches on sport nutrition science have put the focus on the following three groups: ‘muscle mass gain and dietary supplementation’, ‘carbohydrate metabolism’, ‘oxidative stress and dietary supplement use’. The greatest scientific impact can be ascribed to the following topics: ‘nutritional strategies and human skeletal muscle’, ‘dietary supplementation of nitrates’, ‘body weight management’, and ‘gut microbiota’.

**Conclusions:**

Scientific output on sport nutrition has continuously been rising between 2000 and 2018. The ratio of topics related to sport nutrition but predominantly connected to basic research has decreased significantly within all publications. The results of this study confirm the role of science mapping in the identification of specific research topics and primary research directions in the field of sport nutrition science.

**Supplementary Information:**

The online version contains supplementary material available at 10.1186/s12970-021-00409-5.

## Background

Sport nutrition science lies at the intersection of numerous vast fields of life sciences, such as nutrition, clinical, medical, health, biomedical, sport, and food sciences. Sport nutrition science involves the transfer of knowledge related to physical activity and health, metabolism, body composition, diseases, training, injuries, rehabilitation and performance. Sport nutrition as a research topic has attracted great attention in the scientific literature in the field of sport and exercise science. Various systematic reviews or meta-analyses have been conducted on numerous aspects of sport nutrition because of its complex nature that gains interest worldwide [1–6]. Apart from the classical and systematic literature reviews and meta-analyses of the field, there is no large-scale, bibliometric analysis of sport nutrition science. For the experts and sport nutrition practitioners working with athletes, the application of scientific knowledge is essential because of the vast range, dynamism, and multidisciplinary nature of this knowledge output. It is inconceivable to process and apply all of this knowledge without the help of modern information science methods built on international literature databases. These serve as indispensable complementary tools to the standard, literature-synthesizing methods, such as the systematic review and meta-analysis. In the hierarchy of the reliability/quality of scientific results (along with the so-called ‘evidence pyramid’) in sport medicine and nutrition science, systematic reviews and meta-analyses with the processing method of randomized, controlled trials (RCTs) – lies at the top of the pyramid [7]. Science mapping can play an outstanding role alongside meta-analysis to map the knowledge base of the field of sport nutrition science. Science mapping is a type of analysis that usually investigates the structure of literature databases with several thousand-, ten-, or hundred thousand of items covering a long period of time [8]. Applying the statistical-network theoretical modeling of the referencing, text-similarity, and authorial relations of the literature, science mapping methods facilitate the exploration of the conceptual- thematical structure, trends and dynamism of the field of science. Thus, the results of science mapping contribute to strengthening the reliability of scientific results [9, 10].

In sport science, bibliometric analysis has been conducted for the assessment of research trends and for the identification of publications with high impact on the fields of sport psychology [11, 12], sport management [13, 14], sport economy [15], and aging and physical activity [16], however, the analysis of the trends in sport nutrition science has not been performed to date.

## Methodology

### Research aims

This present research on the field of sport nutrition science includes (1) the identification of relevant and state-of-the-art literature, (2) the analysis of trends, key topics and key issues in sport nutrition science, and (3) the identification of the recent trends of the key topics in the time period of 2000–2018. Our research is the first to present the key topics of the publications in sport nutrition science of the past 18 years and their scientometric characteristics identified with the help of bibliometric tools (through the analysis of reference networks and text mining).

### Data collection

**Fig. 1:**
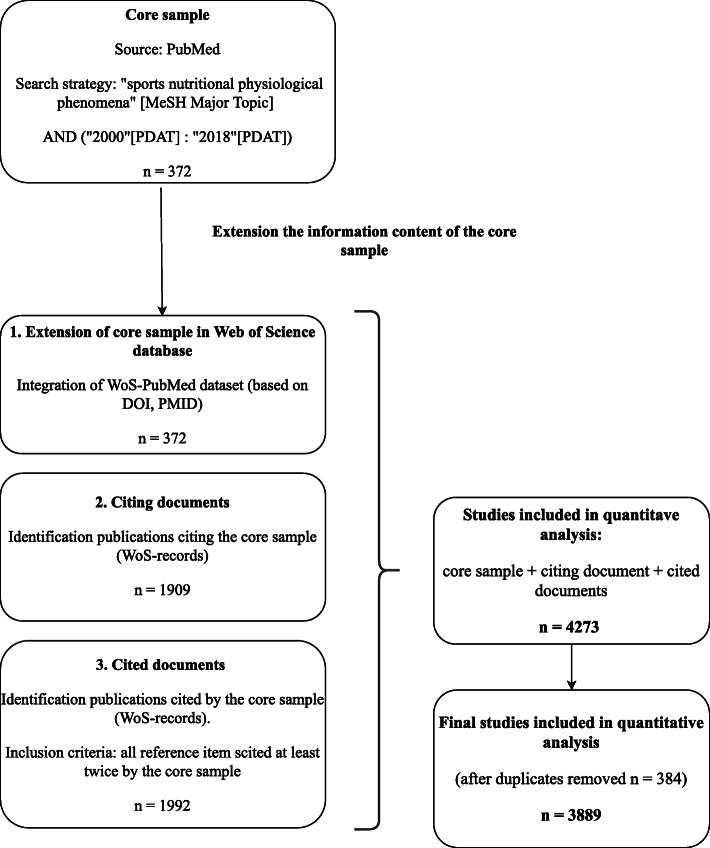
Flowchart of database construction

The core sample (basic documents of the data collection) were obtained from the PubMed medical database with a search from MeSH using the key concepts in “sport nutrition and sport physiology” for the 2000–2018 time period (no results could be retrieved preceding the year 2000 based on this search and via related MeSH major topics). Data was retrieved during February 2019. The search resulted in a core sample of 372 publications. To extend the information content and the time window of the core sample, we used the Web of Science (WoS) database. As a first step, we identified the articles citing the core sample based on WoS records, then we incorporated them into the database (*n* = 1909). Alongside those citing the core, we also identified the publications cited by the core sample (*n* = 1992). Publications were included in the corpus if they received a minimum of two references from the core sample, thus ensuring the inclusion of relevant topics only. After eliminating duplications, the full corpus included 3889 publications, ranging between 1976 and 2018 (Fig. 1).

After identifying the publications in the full corpus (data collection) we continued with their scientometric evaluation based on citation impact and journal rank. To analyze the citation impact, we obtained the impact indicators belonging to the publications of the full corpus from the InCites database, then we expanded the database with the normalized citation impact and the percentile measures. The rank indicators of the journals that include the publications in the full corpus were obtained from the InCites and Journal Citation Report (JCR) databases, after which we expanded the database with the citation quartile based on impact factor.

### Mathematical-statistical analysis

**Fig. 2:**
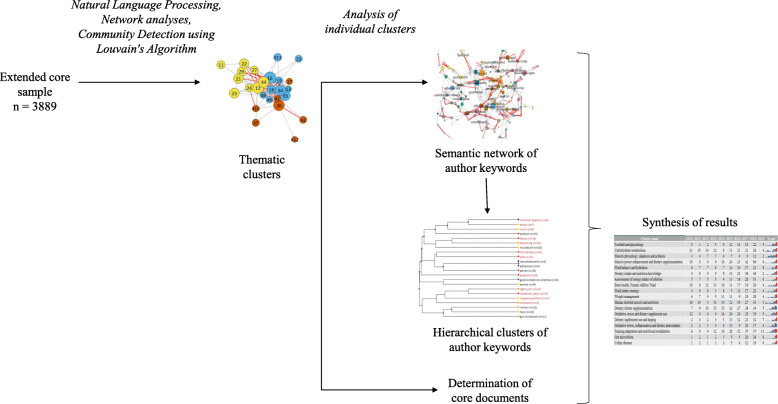
Schematic representation of the analytical framework

The resulting literature corpus was explored through a multi-step methodology combining text mining and bibliometric processes (Fig. 2), which aimed at mapping the structure, trends, key issues and trends of sport nutrition science.

The applied methodology comprised of three parts:

#### Identification of topics

The thematic similarity of publications was explored through the text-similarity of title words and abstracts. With the Natural Language Processing (NLP) method, the compounded text of the titles and abstracts was reduced to key terms that are characteristic for the articles and was represented as the sequence or vector of the frequency of these terms in the articles [17]. Then, a similarity matrix of articles (vectors) was obtained. To demarcate the topic clusters based on article similarity, we derived a graph from the similarity matrix, which we thus considered as a weighed network describing the similarity relations of the publications based on Cosine similarity:

$$ \frac{\sum_{i=1}^n{x}_i{y}_i}{\sqrt{\sum_{i=1}^n{x}_i^2{\sum}_{i=1}^n{y}_i^2}} $$

That is the similarity between article x and y, where x_i_ (y_i_) is the weight of term i for article x.

The actual clustering was achieved by running a community detection algorithm on this proximity network constructed via network text analysis in order to delineate coherent groups of papers. The algorithm used was the “Louvain method”, implemented in the igraph R package [18].

#### The exploration of the key issues, key concepts, and relations of topics

The inner structure of theme clusters and their thematic relations were explored via network analysis. When modeling the relations of key concepts, we defined the network of the joint occurrence of the so-called author keywords assigned to the articles in the cluster, where the strength of the relation between concepts is characterized by the relative frequency of their co-occurrence (among the keywords of the same article). In this case, it is represented by the Cosine similarity as the measure of proximity (this time between concepts, not between articles). This semantic network obtained for the cluster was then subjected to the community detection procedure referred to in the previous section, i.e. the Louvain method [19]. As a result, the network of key concepts was partitioned into cohesive subtopics defined by strongly and densely related concepts.

To gain a more clear representation of conceptual relations, we analyzed the semantic network along different lines as well. We subjected the proximity network (as a proximity matrix) to a hierarchical cluster analysis method (after converting similarities into distances for that end and using the “average” method). The main aim of this additional exercise was to obtain an overview of the domain, where the interrelations of the most relevant concepts are analytically (not only visually) demonstrated by the concept tree resulted from the hierarchical clustering. We call this the “tree view” of the cluster.

In order to capture the conceptual pool most indicative of topics, the procedure described above was run over a selected subset of keywords pertaining of a cluster. The selection of this subset comprised of two steps: (A) the concepts accounting for the cohesiveness of the cluster was identified, using a measure of network role and position, namely, betweenness centrality (BC). BC has been fairly well-validated as measuring the extent to which a particular node (keyword) connects relatively distinct groups (of keywords, i.e. subdiscourses). Applying this measure, keywords with a BC value above a distinctive cutpoint were selected for inclusion in characterizing the cluster (these are the very terms displayed on the dendrograms). (B) For the assignment of key themes and the purposes of labelling, the most frequent words were selected from this pool. This procedure resulted in using a double constraint for keyword relevance: the terms characterizing the cluster had to be both central (in the network sense) and frequent enough for inclusion. Central terms entered into hierarchical clustering, frequent (and central) words were used in cluster labelling and topic designation.

#### The identification of the most important publications (core documents)

For the comprehension and expert characterization of topic clusters, we applied a modern bibliometric method, which algorithmically identifies the most characteristic publications of the topic. The method is the so-called ‘core documents’ methodology [20], the essence of which is the identification of documents that show the biggest possible thematic similarity to the biggest possible segment of the theme (defined by a similarity threshold). More precisely, the identification of core documents was based on the document similarity matrix used for delineating the 18 clusters (recall that his matrix was based on the abstract-, title- and keywords-based (composite) similarity of documents, with values ranging between 0 and 1, standing for complete thematic dissimilarity (0) to full thematic similarity (1), respectively). In this framework, core documents were defined by their thematic similarity to all other cluster members (using a parameter or threshold values chosen experimentally and “Similarity(.,.)” indicating the similarity measure):

D is a core document to Cluster [i] = Min. 50% of P papers in Cluster [i] satisfy the following condition: Similarity(D, P) > 0.6.

We created the list of the identified characteristic publications for each cluster along with the description of the cluster.

## Results

In the course of clustering, we identified four basic groups, from which we highlighted the most relevant 18 topics.

We present the 18 topics according to the following four aspects: the number of publications in the cluster (the size of the topic), the distribution of the publications based on publication date, the citation impact of the publications, and the analysis of the journal rank of publications within each topic.

The 18 topics are the following:

1. Soccer and physiology
2. Carbohydrate metabolism
3. Muscle physiology: alkalosis and acidosis
4. Muscle mass gain and dietary supplementation
5. Fluid balance and hydration
6. Dietary intake and nutrition knowledge
7. Determination of energy need of athletes
8. Bone health, female athlete triad
9. Hydration strategy
10. Body weight management
11. Nutritional Strategies and human skeletal muscle
12. Dietary supplementation of nitrates
13. Oxidative stress and dietary supplement use
14. Dietary supplement use and doping
15. Oxidative stress, inflammation, and dietary antioxidants
16. Exercise adaptation and nutritional strategies
17. Gut microbiota
18. Celiac disease

### Scientometric characteristics of topics

#### The size distribution of topics

**Fig. 3:**
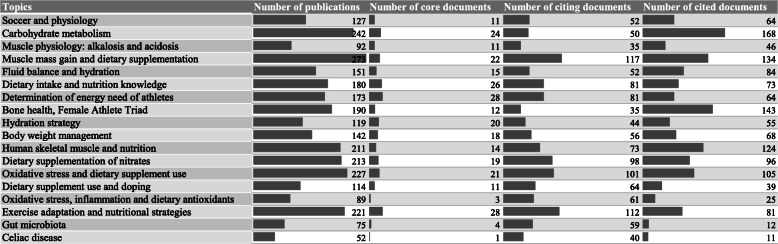
Size distribution of topics

Based on the absolute and relative size of the topics, that are based on the number of publications belonging to a cluster, we can get an insight into the significance of the explored topics and research trends in sport nutrition science. These data are displayed in Fig. 3., which, besides the number of publications in each cluster, also shows their composition in terms of the core publications identified through the targeted search as well as of the publications citing, and those cited by the core sample. In terms of weight (size), the most dominant topic, with ca. three hundred publications, is 'muscle power enhancement and dietary supplementation', followed by topics with about 200–250 publications: ‘carbohydrate metabolism’, ‘oxidative stress and dietary supplement use’, ‘exercise adaptation and nutritional strategies’, ‘dietary supplementation of nitrates’, ‘nutritional strategies and human skeletal muscle’. This group is closely followed by the topic of ‘bone health, female athlete triad’, ‘dietary intake and nutrition knowledge’, and ‘determination of energy need of athletes’, with about 200 publications. The group with 100–150 publications is led by the topics of ‘fluid balance and hydration’, and ‘body weight management’, moreover, here belong the themes of ‘soccer and physiology’, also the ‘hydration strategy’, as well as ‘dietary supplement use and doping’. In the 50–100 range, the dominant topics with almost 100 publications are ‘muscle physiology: alkalosis and acidosis’, ‘oxidative stress, inflammation and dietary antioxidants’, also here belong the smaller but well-defined topics of ‘gut microbiota’ and ‘celiac disease’. The full size of the topics greatly correlates with their role and importance in the core sample; that is, size ranking also reflects the extent to which the cluster is of ‘sport physiological’ nature.

#### Chronological distribution of topics

**Fig. 4:**
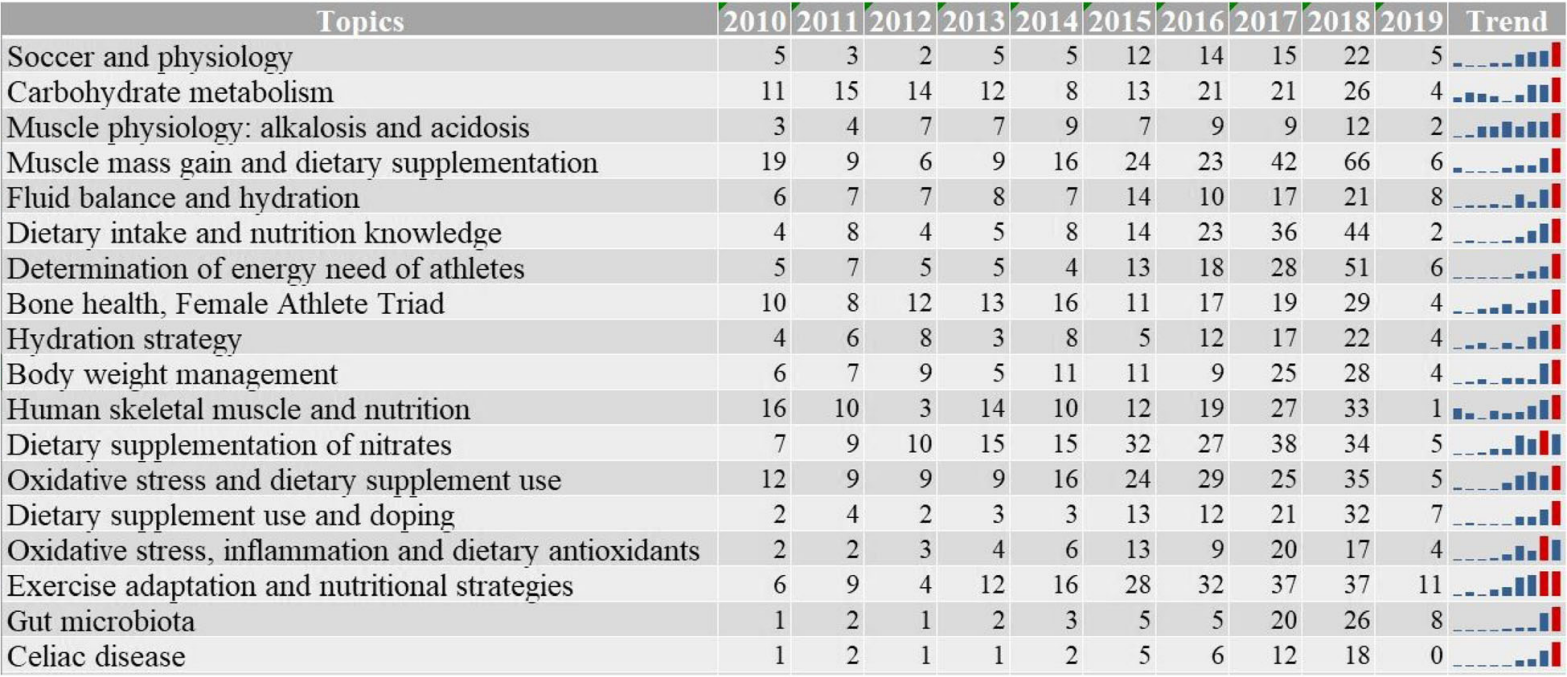
Chronological distribution of topics

The distribution by publication date of the publications belonging to the identified topics, or, to put it another way, the chronological distribution of each cluster facilitates the assessment of the trends and relevance of each topic, of their ‘popular’ or ‘winding down’ nature. The distribution of the size of the topics is summarized in Fig. 4., which shows the number of documents belonging to each topic starting from 2010. Based on this, the trend graphs in the table provide an overview of the chronological dynamics of the topics. The trend graphs attest that each topic shows a growing tendency (that is, more and more articles appeared on the given topic in the past decade), each cluster is at its maximum size in the last examined year (2018). The differences between the topics appear in the characteristics and pattern of the increase. According to the latter, with a little simplification, we observe two types of increase: (1) the topic increases gradually from the beginning or middle of the examined decade, (2) after a relative ‘stagnation’, the topic shows a rising slope in the last few years. Type (1), a gradual increase is the most characteristic of the topics ‘muscle physiology: alkalosis and acidosis’, ‘nutritional strategies and human skeletal muscle’, ‘exercise adaptation and nutritional strategies’. Type (2), sudden increase with a more moderate rising slope, spread over the last 4-5 years, appears in relation with the topics ‘soccer and physiology’, ‘energy intake and nutrition knowledge’, ‘nutritional strategies and human skeletal muscle’, ‘fluid balance and hydration’, ‘hydration strategy’, ‘oxidative stress and dietary supplement use’, ‘dietary supplement use and doping’, ‘oxidative stress, inflammation and dietary antioxidants’. In the case of topics with a Type (2) the slope is rising and normally peaking in the last 2 years, these are the following: ‘muscle power enhancement and dietary supplementation’, 'determination of energy need of athletes’, ‘body weight management’, ‘gut microbiota’, and ‘celiac disease’. A ‘unique’ cluster, with a somewhat different pattern from the two basic types is the ‘carbohydrate metabolism’, which, along with some fluctuations, shows a steady output in the topic (also with a slight increase); another is ‘bone health and female athlete triad’, which also shows a steady output with a rising slope in the last examined year.

#### Citation impact of topics and journal ranks

Apart from chronological trends, the relevance and scientific significance of topics can be investigated through the use of the following two groups of scientometric indicators: (1) the citation measures (of the publications) of individual topics, and (2) the prestige or rank of publication venues, i.e., journals. Citation measures provide information about the scientific impact exerted by publications within a topic, considered as a fundamental proxy to the relevance of topics for sport nutrition science.

**Fig. 5:**
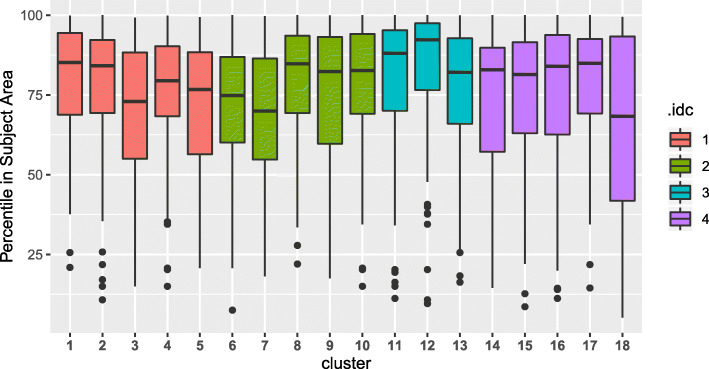
The distribution of normalized citation impact (citation percentile) for clusters

Instead of the raw citation number, correcting for the field- and age-related differences, publications are characterized by their position in the citation distribution within the particular research field, i.e. with the citation percentile. The clusters with the biggest impact are those whose average, that is, the characteristic value of the relevant articles is situated closest to 100. Those with a median above the value of 75 can be considered to have a high general citation impact (they belong to the most cited, 25%, citation quartile). Based on this, practically all the identified clusters fall into the high citation measure range, both in terms of their median and of the majority (minimum 50%) of their publications. The topics with the biggest impact are the ‘nutritional strategies and human skeletal muscle’ and ‘dietary supplementation of nitrates’. A similarly high composite impact is shown by the topics of ‘soccer and physiology’, ‘carbohydrate metabolism’, ‘bone health and female athlete triad’, ‘body weight management’, and ‘gut microbiota’ (Fig. 5).

**Fig. 6:**
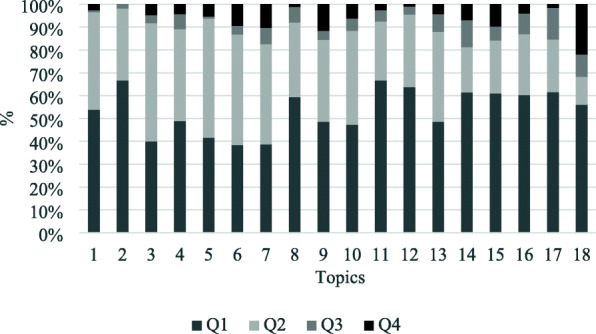
The rank of publications in the citation quartile system

The rank of publications, in this case, refers to the rank and recognition of the publishing journals, with the latter representing, in terms of the topics, the quality of knowledge transferred in them. To describe the rank and the quality of the knowledge content of topics, we applied the citation quartile system. The basis of the approach is the classification of publications into four quality classes according to the rank of the publishing journal. The so-called Q1 journals belong to the upper 25% of the journal rank of the field, Q2 journals belong to the upper 25–50% quartile, Q3 journals are in the lower 25–50%, and Q4, in the bottom 25%. Figure 6. shows the distribution of the articles of the identified clusters among the four classes for each theme. In this case, also, we can state that almost all clusters include publications of high quality, inasmuch as on average, 50% of their publications are ranked Q1, and the majority of the rest of the publications belong to Q2, which can also be considered a satisfactory quality rank. ‘Carbohydrate metabolism’, ‘nutritional strategies and human skeletal muscle’, ‘dietary supplementation of nitrates’ stand out (Fig. 6).

In the next step of our methodological process, we applied the scientometric assessment of topics, for which we selected the four most significant topics and presented their internal structure in terms of key thematic relations based on our text mining and core document methodologies. We illustrated the conceptual network of topics with a dendrogram, and we analyzed the four topics based on their interconnections from the aspects of sport nutrition, sport physiology, type of sport, dietary supplements, and performance diagnostics. The collection of the 18 topics and their core papers are in Additional file 1. Using an advanced bibliometric method, we obtained the so-called core papers for each research topic, the most representative publications in the cluster (see Additional file 1).

##### Description of the nutritional strategies and human skeletal muscle cluster

**Fig. 7:**
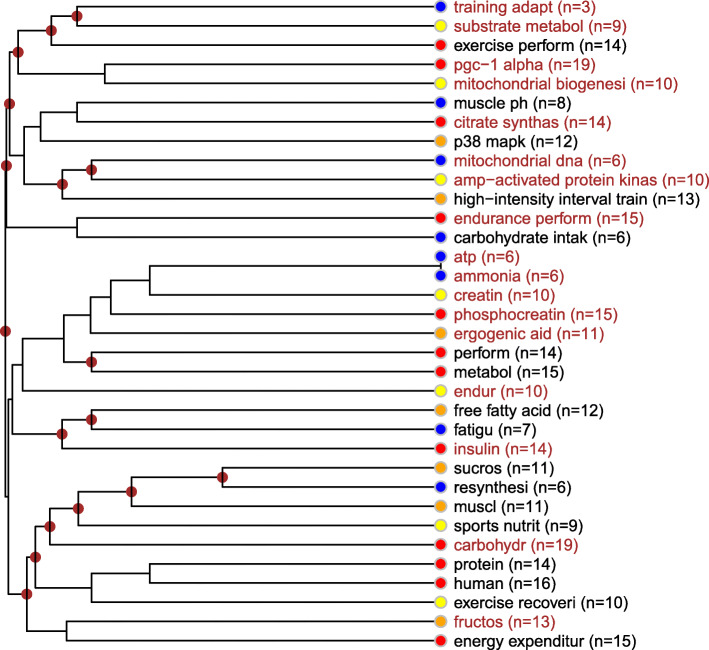
Nutritional strategies and human skeletal muscle topic. Notes: The keywords and concept groups belonging together are displayed in the same font color. Next to each keyword, color codes designate the occurrence frequency and importance of the given concept within the topic (color legend: red: high frequency, belonging to the top citation quartile of the distribution; orange: frequent, belonging to the third citation quartile of the distribution; yellow: medium frequency, belonging to the second citation quartile of the distribution; blue: low frequency, belonging to the bottom citation quartile of the distribution).

This topic focuses on the relationship between sport performance and the metabolic pathways of skeletal muscle cells. Its most frequent issue is the role of PGC-1 alpha transcriptional regulation especially in the relation to mitochondrial biogenesis, citrate synthesis, endurance performance, phosphocreatine as the creatine substrate of performance enhancement, the relation of insulin, free fatty acids and fatigue, protein and carbohydrates (first of all fructose), and also in relation to energy expenditure and regeneration. Depending on the type of the examined sport, endurance training stands out, but high-intensity interval training is also present with almost the same frequency. Of the biological components, the role of P38 MAPK protein kinase is worth emphasizing (Fig. 7).

##### Description of the dietary supplementation of nitrates topic

**Fig. 8:**
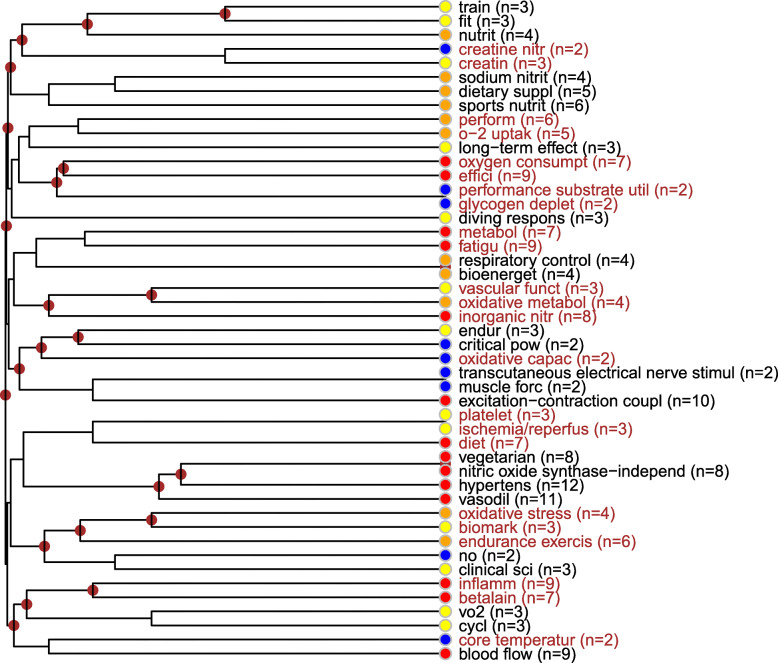
Dietary supplementation of nitrates topic

The complex (sport-) physiological focus of the topic is on the relation network of blood circulation, oxidative metabolism and nitrogen metabolism and supplementation (in relation to sport activities). Its primary topics are oxygen consumption (related to the concept of efficiency), fatigue, inorganic nitrate substrate related to vascular function, the stimulus-muscle contraction connection, vegetarianism, the enzyme that produces nitrogen oxide substrate, the relation network of hypertension and vasodilatation, inflammation and beta-alanine, and bloodstream. Depending on the type of physical activity, this topic is characterized by endurance training, especially, related to oxidative stress and its biomarkers. The concept of diving response also emerged mainly related to oxygen uptake and consumption and more slightly to respiratory control. Within further substrata, sodium nitrite and with lower frequency, creatine-nitrate appeared. Regarding indices, the use of the VO2 performance indicator was relevant (Fig. 8).

##### Description of the carbohydrate metabolism topic

**Fig. 9:**
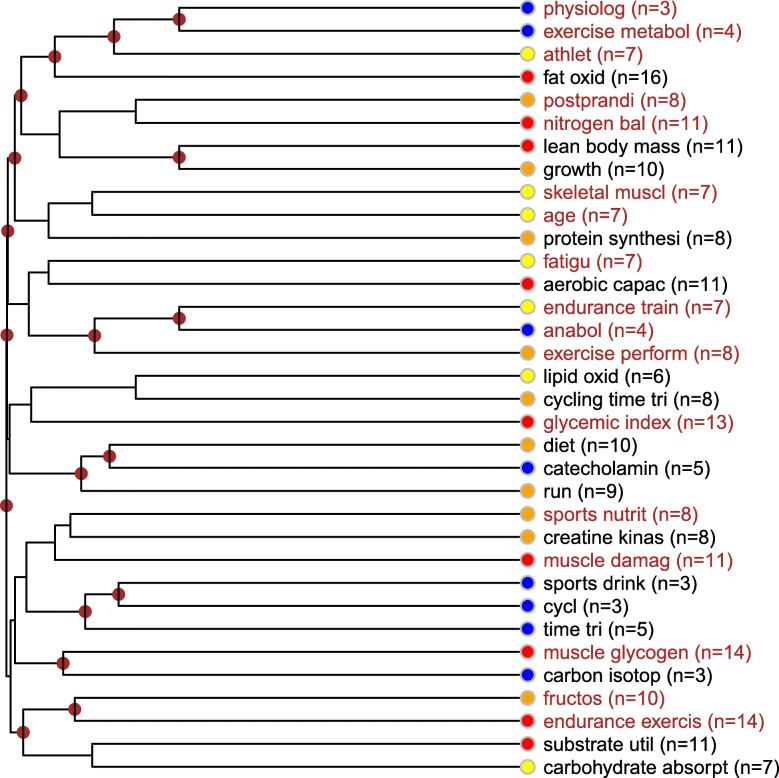
Carbohydrate metabolism topic

From a sport physiological point of view, this topic also revolves around the improvement of skeletal muscle function, mainly in the relation to carbohydrate metabolism. Its pivotal concepts and issues are lipid oxidation during exercise metabolism processes, nitrogen balance (especially after eating), lean body mass (increase), aerobic capacity related mostly to fatigue and endurance training, glycemic index, muscle glycogen, and endurance training (and carbohydrate absorption). The latter is characteristic of the type of the examined sport and in the terms of branch of sport, cycling (timed training) and running emerged. In the case of the examined substrates, dietary supplements and biologically active components, the role of lipid oxidation (timed cycling), and - less frequently - catecholamine (running), sport drinks (cycling) and fructose (endurance training), and creatine kinase emerged (Fig. 9).

##### Description of the muscle mass gain and dietary supplementation topic

**Fig. 10:**
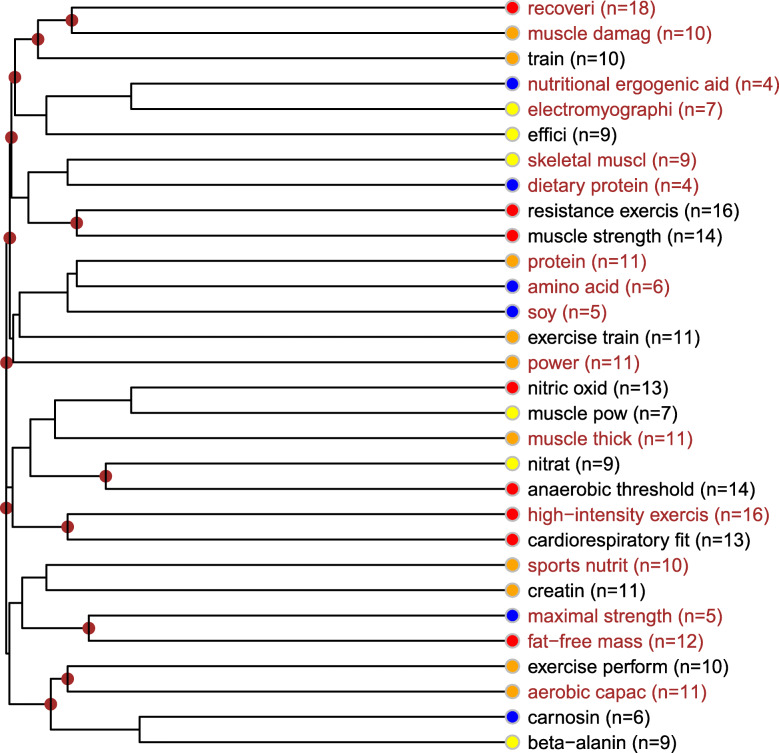
Muscle mass gain and dietary supplementation topic

The central topics of this cluster are the factors of muscle mass gain and strengthening. The most important questions of these topics - that the studies focus on - are the association between muscle recovery and injuries, muscle strength, anaerobe threshold, anaerobe capacity, cardiorespiratory endurance, and lean body mass. Categorized by the type of sport exercise the two major topics are resistance exercise and high-intensity exercise related mostly to cardiorespiratory endurance. The topic of dietary supplement use is also significant in this cluster. Proteins are the most important and nitrogen oxides, nitrates and creatine are also considerable among the studies on substrates and dietary supplements that play an important role in muscle mass gain and performance enhancement. Carnosine and beta-alanine have a relatively smaller significance in this topic. In this cluster, electromyography is a frequent research method (Fig. 10).

##### Nutrients and substrates that appeared in the clusters

Among the macronutrients, proteins and carbohydrates appeared the most frequently in the topics. These two nutrients were studied according to their general role in nutrition and also as ergogenic nutrients in sport nutrition. The role of proteins and amino acids were assessed in the ‘muscle mass gain and dietary supplementation’ and the ‘oxidative stress, inflammation, and dietary antioxidants’ clusters. In the carbohydrate metabolism cluster - more specifically - glucose plays a major role in the ‘soccer and physiology’ and fructose in the ‘nutritional strategies and  human skeletal muscle’ clusters. Regarding further nutrients and substrates, beta-alanine, creatine, caffeine, nitrates, and sodium appears frequently in the analysed publications. The effect of beta-alanine was studied in the ‘muscle physiology: alkalosis and acidosis’, ‘exercise adaptation and nutritional strategies’ and the ‘muscle mass gain and dietary supplementation’ clusters. The role of nitrates was assessed in the ‘dietary supplementation of nitrates’ and in the ‘oxidative stress and dietary supplement use’ clusters. Besides the nitrates, caffeine was also analysed in these two latter clusters. Furthermore, the topic on the effect of caffeine had a major role in the ‘determination of energy need of athletes’ and ‘muscle physiology: alkalosis and acidosis’ clusters. The effect of creatine was studied in the ‘body weight management’, ‘muscle mass gain and dietary supplementation’ clusters. The pre- and probiotics were only assessed in the ‘gut microbiota’ cluster. The role of omega-3 fatty acids was analysed as stress and inflammatory marker in the ‘oxidative stress and dietary supplement use’ cluster (Table 1).

**Table 1:** Nutrients and substrates appeared and analyzed in the clusters

Topic	Nutrients and substrates
soccer and physiology	carbohydrates, glucose, anthocyanin
carbohydrate metabolism	proteins, carbohydrates, glucose, fructose
muscle physiology: alkalosis and acidosis	sodium-bicarbonate, carnosine, caffeine, beta-alanine,
muscle mass gain and dietary supplementation	creatine, beta-alanine, proteins, amino acids,
fluid balance and hydration	carbohydrates, sodium-bicarbonate, proteins
dietary intake and nutrition knowledge	beta-alanine, proteins
determination of energy need of athletes	carbohydrates, proteins, caffeine
bone health, female athlete triad	calcium
hydration strategy	carbohydrates, sodium-bicarbonate
body weight management	proteins, creatine
nutritional strategies and human skeletal muscle	carbohydrate, protein, sodium- bicarbonate, fructose
dietary supplementation of nitrates	dietary nitrates, beetroot juice, caffeine
oxidative stress and dietary supplement use	antioxidants, creatine, omega-3 fatty acids, vitamin E,
dietary supplement use and doping	creatin, caffeine, nitrate
oxidative stress, inflammation and dietary antioxidants	antioxidants, green tea, carnosine, beta-alanine, amino acids
exercise adaptation and nutritional strategies	carbohydrates, fats, beta-alanine
gut microbiota	protein, probiotics
celiac disease	creatine, sodium

## Discussion

The results of the present study confirm the role of science mapping in (1) the identification of specific research topics, primary research directions, and (2) the exploration of the relative significance of research directions, their spatial and temporal dynamism. These factors facilitate the more precise identification of major research directions, the optimization of available research resources, and the implementation of developmental research more focused on actual needs.

**Fig. 11:**
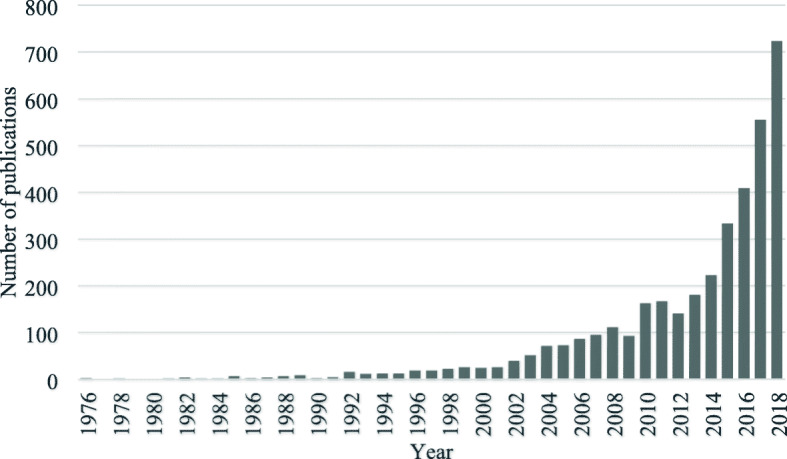
Distribution of sample papers by publication year

In our search strategy to delineate the scientific discourse under study we employed a two-stage model, where first an expert-based core is outlined (PubMed search via MeSH), which, in turn, is extended by bibliometric methods, namely, the citation environment of the core. This procedure had profound implications on the time window under study: the PubMed database provided a sample ranging from the 2000’s to date, while the bibliometric extension, the augmented or full sample, resulted in a much longer time period, spanning between 1976 and 2018. Hence, this latter period was the true interval for our sample. However, analysis of the distribution of sample papers by publication year (Fig. 11) demonstrated that it is from the 2000’s that a statistically meaningful amount of publications appeared in the sample, accompanied with a rapid growth from around this year. Consequently, our results in fact reflect the structure of the field between 2000 and 2018, despite the much broader ‘nominal’ coverage of the sample. We can therefore set this period as the time window for our analysis and results, being highlighted in the title of the paper. Also, we conducted a statistical analysis of the trend of publication growth, identifying so-called ‘breakpoints’ that indicate substantial changes in the trend [21]. It also confirmed that our mapping has practically been focused on this 20-year time period (see Additional file 2).

**Fig. 12:**
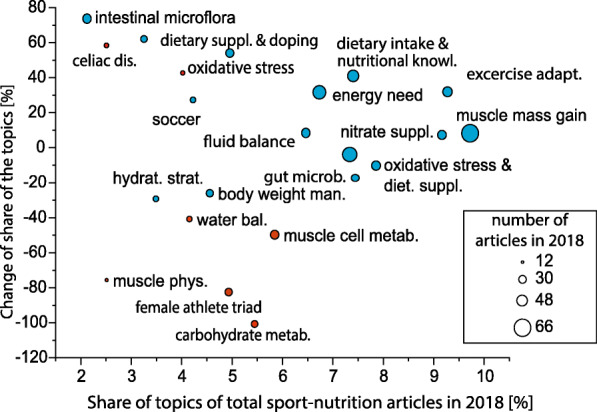
BCG matrix of sport nutrition research trends. (color legend: red: topics related to basic research, blue: main directions of applied researches)

To illustrate the outlined connections, we present the results of our research with the help of the Boston Consulting Group (BCG) matrix, which is widely applied in management practice [22]. This visualization technique operates a coordinate system with one axis showing the market share (in our case: the ratio within all sport nutrition publications) and the other axis showing the rate of change over the examined period of time (2010–2018), (Fig. 12).

The Fig. 12. attests to the great diversity of topics related to sport nutrition: no topic has a ratio higher than 10 % among all publications related to sport nutrition. The number and ratio of publications on ‘muscle power enhancement and dietary supplementation’ are relatively stable. Besides, there is a consistent focus on the topics of ‘dietary supplement use and doping’ and the issue of ‘dietary supplementation of nitrates’. Over the past decade, the number of publications about the association between ‘dietary supplement use and doping’ and those on ‘nutrition knowledge of athletes’ has shown the most considerable increase.

A rising slope as a tendency was observed in the significance of researches on the nutrition of athletes with celiac disease and the importance of discipline-specific (e.g. soccer) analysis is also increasing. Notably, the ratio of topics related to sport nutrition predominantly connected to basic research (e.g. ‘muscle physiology: alkalosis and acidosis’, ‘carbohydrate metabolism’) has significantly decreased within all publications. This could be due to the fact that existing knowledge in sport nutrition science has become suitable for offering practical applications (e.g. development of dietary supplements) over the past decade. Based on Fig. 12, four facts can be stated related to the main directions of development in the future:

1. Basic research continues to have a significant role as it provides the scientific foundation of specific product development.
2. Sport nutrition analyses related to the nutritional awareness of athletes and the restriction of doping substances will have increasing importance.
3. Special nutritional needs (e.g., food intolerance, vegetarianism, observation of religious regulations such as halal products) will have growing relevance in sports.
4. The importance of nutrition research on the unique needs of specific types of sport will also be increasing.

Several systematic reviews and position stands have been written in the field of sport nutrition and the most frequent topics in these publications are the following: creatine, protein, probiotics, beta-alanine and caffeine supplementation regarding different aspects of exercise [23–26]. Nutrition knowledge of athletes, body composition and nutrition of young female athletes are also highlighted topics of reviews [27–29]. Based on the results of the bibliometric mapping, all of these themes appear in the clusters and present the aspects of sport nutrition from a new perspective. In fact, nutrition of female athletes is the key-question of the ‘bone health, female athlete triad’ topic and body composition is the core of ‘muscle mass gain and dietary supplementation’ or ‘body weight management’ topics. The main topics of sport nutrition systematic reviews and position stands are the basis of the clusters mentioned and described above.

In the sport nutrition review of Kerksick et al. (2018), ergogenic nutrients and dietary supplements that are taken by athletes were categorized into three categories: strong evidence to support the efficacy and apparently safe; limited or mixed evidence to support the efficacy; little to no evidence to support the efficacy and/or safety. They emphasized that ‘evaluating the available scientific literature is an important step in determining the efficacy of any diet or the dietary supplement' [2]. Thus, their categorization of dietary supplements was based on the available scientific literature. Most muscle building and performance enhancement supplements that appeared in the clusters were classified into the ‘strong evidence to support efficacy and apparently safe’ category in the work of Kerksick et al. (2018). We can state that our results fit with the research trends of sport nutrition and the systematic synthesis and bibliometric analysis of the available scientific literature are complementary to each other.

Our study - like all of the literature reviews and bibliometric mapping study - is a necessary compromise between the limits of resources, time-frame of research, and length of publication. That’s why some limitations of the current study should be highlighted. (1) The pool of publications, used for our research: we have applied the two most prestigious databases of nutrition and medical and health sciences: the Pubmed and the WoS. The involvement of other databases (e.g. Scopus) and another language (e.g. Chinese) could further increase the set of information suitable for analysis. (2) We have to take into consideration that the relation between nutrition and physical performance has a very important and direct practical relevance. That’s why the published results of scientific research not necessarily reflect the frontiers of the actual knowledge, because the interests of business (e.g. pharmaceutical and/or nutraceutical companies), sport (e.g. Olympic teams), as well as military and law enforcement organizations (e.g. the analysis of acknowledgment section of articles shows that one of the main sponsors of – rather cost-intensive research activities – are the armies) often limit the content of published information. A further limiting factor is related to the search strategy. (3) We applied a major MeSH term as a search query instead of creating a detailed search algorithm. (4) We tacitly supposed that the abstract exactly reflects the key points of the research goals and results. It is possible that this assumption was biased because there are some difference between the content of the abstract and that of the text of articles. (5) Last, but not least, we should note that bibliometric indicators (as basically all kinds of indicators) have their limitations and validity issues. Whether and in what sense citation indicators convey quality, impact, relevance, importance is a matter of active scholarly debate and theorizing since the advent of professional scientometrics. Beyond controversies, however, (1) various theories of citation (and empirical studies) converge on the conclusion that despite the uncertainties and accidental features of individual citations, at the aggregate and statistical level, citation measures can and, if well selected and employed, do have construct validity to scientific impact. Moreover (2) evaluative citation analysis is a legitimate and, to a fair extent, validated methodological framework for quantitative studies of science, which we chose as our framework.

**Fig. 13:**
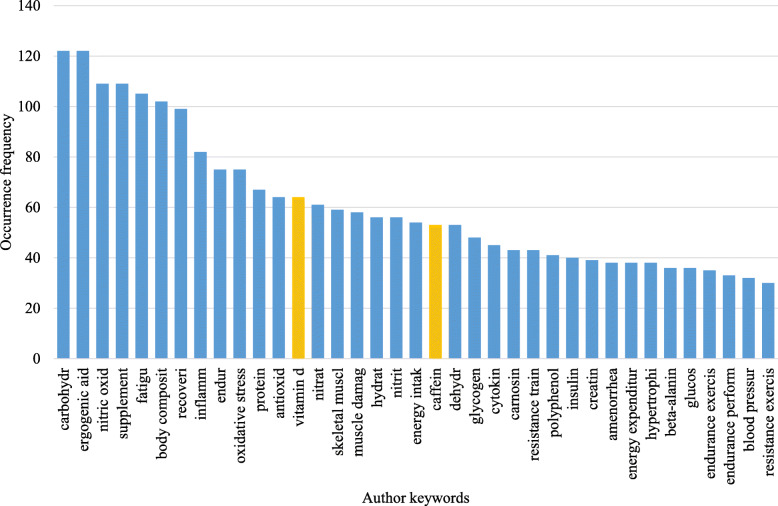
The top-end of the frequency distribution of keywords in the sample corpus

Regarding our complete methodology, it is very important to emphasize that the hidden strategy behind bibliometric mappings is to complement or contrast the expert’s interpretation of a research field with the interpretation based on scholarly communication. Both are equally valid and valuable, but the latter may represent a somewhat different unit of the field and enrich the experts’ conceptualization. Precisely, in our case, the sample was based on a field-specific MeSH Major Topic as the expert-based “core” and was extended by discovering its citation environment in WoS. Thus, keywords plus citation relations that were used, which may go beyond a field-specific journal set, or even cross-classify the journal-based literature. However, it was our intention: we were interested in portraying the hidden units of sport nutrition research that emerges from scholarly communication within a preselected time window (from 2000 on). The same principle applies to topic formation: we aimed to uncover hidden relations (and clusters), which may not coincide with the most important and extensive theme categories. Furthermore, size (publication count) was only one aspect of excellence (beyond impact, quality, novelty, etc.) we used for characterizing topics. To illustrate this (whole) point, we added a figure showing the top-end of a simple frequency distribution of keywords in our sample, which does not reflect conceptual relations and structure (as opposed to our clusters). We found vitamin D and caffeine as relatively frequent topics, both known and indicated to be prominent concepts in the - long term - development of the field, but both being distributed among topic clusters where conceptual relations are being targeted (Fig. 13).

## Conclusions

Our research is the first large-scale bibliometric mapping study that presents the key topics of the publications in sport nutrition science of the past 10 years. In the course of the bibliometric analysis of sport nutrition science research, we analyzed 3889 scientific publications, which provided an insight into the most relevant research trends, the scientific impact of publications, the diachronic distribution of specific research directions, and the rank of publications. Altogether, we were able to identify four main groups. From the four main groups, we analyzed the 18 most dominant topics, from which four are portrayed in detail in the present article.

Based on the size of the topics, research in sport nutrition science has put the biggest emphasis on the three topics of ‘muscle mass gain and dietary supplementation’, ‘carbohydrate metabolism’, ‘oxidative stress, and dietary supplement use’. ‘Exercise adaptation and nutritional strategies’ make up nearly 8% of the full corpus, another 8% is made up of the relationship of performance and nutrition, and the ‘determination of energy need of athletes’, finally, 8% is made up by ‘dietary intake of athletes and nutrition knowledge’. Regarding the prevalence of specific topics, ‘muscle mass gain and dietary supplementation’, ‘determination of energy need of athletes’, ‘body weight management’, ‘gut microbiota’, and ‘celiac disease’ have rapidly become outstanding research topics in the last 2 years. Topics with the greatest scientific impact are ‘nutritional strategies and human skeletal muscle’, ‘dietary supplementation of nitrates’, ‘body weight management’, and ‘gut microbiota’. In terms of the quality of the transferred information, the topics of ‘carbohydrate metabolism’, ‘nutritional strategies and human skeletal muscle’, and ‘dietary supplementation of nitrates’ stand out.

Our research provided a justification for applying bibliometric analysis in four dimensions of the research topic:

- Based on the number of publications in the cluster (the size of the topic), we get an insight into the impact of identified topics and research directions in the field of sport nutrition science.
- The distribution of the publications based on publication date shows the trends and relevance of the specific topics.
- The citation measure of the publications of a topic shows the scientific impact of publications of specific topics, which is a fundamental approach to the sport nutrition science relevance of themes.
- The exploration of the prestige or rank of publications within each cluster: in this case, the rank of publication means the rank or recognition of the publishing journal, with the latter representing, in terms of the themes, the ‘quality’ of transferred knowledge related to the theme.

It can be stated that sport nutrition is a rapidly developing, organically evolving, interdisciplinary field of science. New frontiers of knowledge and challenges of demand force a constantly increasing scope of this science, parallel with continuous deepening of understanding the interplay of different biological and chemical systems with inherent demand of modern sport. Results of the current research highlight the importance of bibliometric mapping studies in this field, because this approach offers an exact way to determine the future, prospective ways of further development and promote the optimal allocation of scarce intellectual and material resources of research.

## Supplementary Information

**Additional file 1.**

**Additional file 2.**

## Data Availability

A part of data generated or analyzed during this study are included in this published article [and its supplementary information files]. The complete datasets used and/or analyzed during the current study are available from the corresponding author on reasonable request.

## Abbreviations

MeSH: Medical Subject Headings
NPL: Natural Language Processing
WoS: Web of Science
JCR: Journal Citation Report
BC: Betweenness centrality
