# Supplementary material for: Structure and trends of international sport nutrition research between 2000 and 2018: bibliometric mapping of sport nutrition science
Source: J Int Soc Sports Nutr. 2021 Feb 5;18:12. doi: 10.1186/s12970-021-00409-5 (PMC7866438; doi:10.1186/s12970-021-00409-5)
Supplement: Supplementary file 1 — Additional file 1. [file 12970_2021_409_MOESM1_ESM.docx]

We identified four basic groups, from which we highlighted the most relevant 18 topics in sport nutrition research. This file gives an overview about the “tree view” of the 18 topics and their core documents (the most representative publications in the cluster).

Notes: The key words and concept groups belonging together are displayed in the same letter color. Next to each key word, color code designates the occurrence frequency and importance of the given concept within the theme (color legend: red: high frequency, belonging to the top citation quartile of the distribution; orange: frequent, belonging to the third citation quartile of the distribution; yellow: medium frequency, belonging to the second citation quartile of the distribution; blue: low frequency, belonging to the bottom citation quartile of the distribution).

**1) Football and physiology**


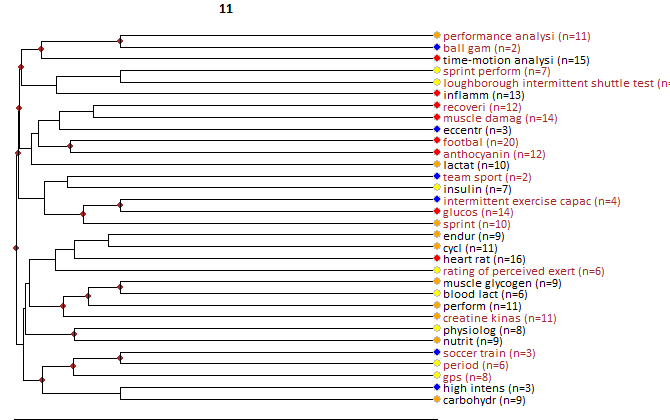


Core documents

| 1. McGregor, SJ; Nicholas, CW; Lakomy, HKA; Williams, C (1999) The influence of intermittent high-intensity shuttle running and fluid ingestion on the performance of a soccer skill. JOURNAL OF SPORTS SCIENCES, 17:11, 895-903. |
| --- |
| 1. Nicholas, CW; Nuttall, FE; Williams, C (2000) The Loughborough Intermittent Shuttle Test: A field test that simulates the activity pattern of soccer. JOURNAL OF SPORTS SCIENCES, 18:2, 97-104. |
| 1. Mohr, M; Krustrup, P; Bangsbo, J (2003) Match performance of high-standard soccer players with special reference to development of fatigue. JOURNAL OF SPORTS SCIENCES, 21:7, 519-528. |
| 1. Bangsbo, J; Mohr, M; Krustrup, P (2006) Physical and metabolic demands of training and match-play in the elite football player. JOURNAL OF SPORTS SCIENCES, 24:7, 665-674. |
| 1. Krustrup, P; Mohr, M; Steensberg, A; Bencke, J; Kjaer, M; Bangsbo, J (2006) Muscle and blood metabolites during a soccer game: Implications for sprint performance. MEDICINE AND SCIENCE IN SPORTS AND EXERCISE, 38:6, 1165-1174. |
| 1. Cunniffe, B; Proctor, W; Baker, JS; Davies, B (2009) AN EVALUATION OF THE PHYSIOLOGICAL DEMANDS OF ELITE RUGBY UNION USING GLOBAL POSITIONING SYSTEM TRACKING SOFTWARE. JOURNAL OF STRENGTH AND CONDITIONING RESEARCH, 23:4, 1195-1203. |
| 1. Magalhaes, J; Rebelo, A; Oliveira, E; Silva, JR; Marques, F; Ascensao, A (2010) Impact of Loughborough Intermittent Shuttle Test versus soccer match on physiological, biochemical and neuromuscular parameters. EUROPEAN JOURNAL OF APPLIED PHYSIOLOGY, 108:1, 39-48. |
| 1. Osgnach, C; Poser, S; Bernardini, R; Rinaldo, R; Di Prampero, PE (2010) Energy Cost and Metabolic Power in Elite Soccer: A New Match Analysis Approach. MEDICINE AND SCIENCE IN SPORTS AND EXERCISE, 42:1, 170-178. |
| 1. Bradley, PS; Di Mascio, M; Peart, D; Olsen, P; Sheldon, B (2010) HIGH-INTENSITY ACTIVITY PROFILES OF ELITE SOCCER PLAYERS AT DIFFERENT PERFORMANCE LEVELS. JOURNAL OF STRENGTH AND CONDITIONING RESEARCH, 24:9, 2343-2351. |
| 1. Russell, M; Benton, D; Kingsley, M (2012) Influence of carbohydrate supplementation on skill performance during a soccer match simulation. JOURNAL OF SCIENCE AND MEDICINE IN SPORT, 15:4, 348-354. |
| 1. Raman, A; Macdermid, PW; Mundel, T; Mann, M; Stannard, SR (2014) The Effects of Carbohydrate Loading 48 Hours Before a Simulated Squash Match. INTERNATIONAL JOURNAL OF SPORT NUTRITION AND EXERCISE METABOLISM, 24:2, 157-165. |
| 1. Funnell, MP; Dykes, NR; Owen, EJ; Mears, SA; Rollo, I; James, LJ (2017) Ecologically Valid Carbohydrate Intake during Soccer-Specific Exercise Does Not Affect Running Performance in a Fed State. NUTRIENTS, 9:1, -. |
| 1. Silva, JR; Rumpf, MC; Hertzog, M; Castagna, C; Farooq, A; Girard, O; Hader, K (2018) Acute and Residual Soccer Match-Related Fatigue: A Systematic Review and Meta-analysis. SPORTS MEDICINE, 48:3, 539-583. |
| 1. Pereira, LA; Nakamura, FY; Moraes, JE; Kitamura, K; Ramos, SP; Loturco, I (2018) MOVEMENT PATTERNS AND MUSCLE DAMAGE DURING SIMULATED RUGBY SEVENS MATCHES IN NATIONAL TEAM PLAYERS. JOURNAL OF STRENGTH AND CONDITIONING RESEARCH, 32:12, 3456-3465. |

**2) Carbohydrate metabolism**


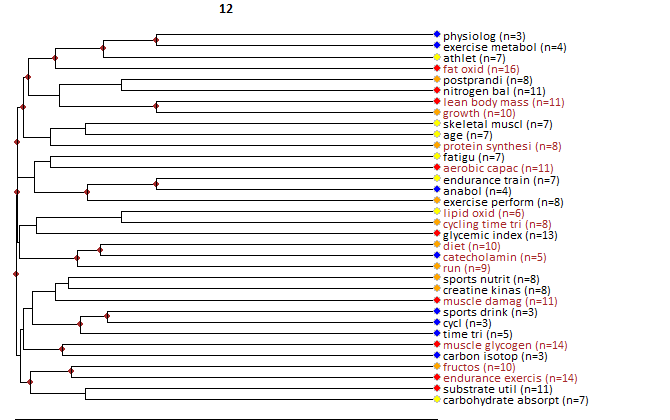


Core documents

| 1. Wee, SL; Williams, C; Gray, S; Horabin, J (1999) Influence of high and low glycemic index meals on endurance running capacity. MEDICINE AND SCIENCE IN SPORTS AND EXERCISE, 31:3, 393-399. |
| --- |
| 1. Pitsiladis, YP; Maughan, RJ (1999) The effects of exercise and diet manipulation on the capacity to perform prolonged exercise in the heat and in the cold in trained humans. JOURNAL OF PHYSIOLOGY-LONDON, 517:3, 919-930. |
| 1. Jentjens, RLPG; Cale, C; Gutch, C; Jeukendrup, AE (2003) Effects of pre-exercise ingestion of differing amounts of carbohydrate on subsequent metabolism and cycling performance. EUROPEAN JOURNAL OF APPLIED PHYSIOLOGY, 88:43560, 444-452. |
| 1. Ivy, JL; Res, PT; Sprague, RC; Widzer, MO (2003) Effect of a carbohydrate-protein supplement on endurance performance during exercise of varying intensity. INTERNATIONAL JOURNAL OF SPORT NUTRITION AND EXERCISE METABOLISM, 13:3, 382-395. |
| 1. Coyle, EF (1991) Timing and method of increased carbohydrate intake to cope with heavy training, competition and recovery. JOURNAL OF SPORTS SCIENCES, 9:, 29-51. |
| 1. Koopman, R; Pannemans, DLE; Jeukendrup, AE; Gijsen, AP; Senden, JMG; Halliday, D; Saris, WHM; van Loon, LJC; Wagenmakers, AJM (2004) Combined ingestion of protein and carbohydrate improves protein balance during ultra-endurance exercise. AMERICAN JOURNAL OF PHYSIOLOGY-ENDOCRINOLOGY AND METABOLISM, 287:4, E712-E720. |
| 1. Chryssanthopoulos, C; Williams, C; Nowitz, A; Bogdanis, G (2004) Skeletal muscle glycogen concentration and metabolic responses following a high glycaemic carbohydrate breakfast. JOURNAL OF SPORTS SCIENCES, 22:43781, 1065-1071. |
| 1. Betts, JA; Stevenson, E; Williams, C; Sheppard, C; Grey, E; Griffin, J (2005) Recovery of endurance running capacity: Effect of carbohydrate-protein mixtures. INTERNATIONAL JOURNAL OF SPORT NUTRITION AND EXERCISE METABOLISM, 15:6, 590-609. |
| 1. Millard-Stafford, M; Warren, GL; Thomas, LM; Doyle, JA; Snow, T; Hitchcock, K (2005) Recovery from run training: Efficacy of a carbohydrate-protein beverage?. INTERNATIONAL JOURNAL OF SPORT NUTRITION AND EXERCISE METABOLISM, 15:6, 610-624. |
| 1. Koopman, R; Beelen, M; Stellingwerff, T; Pennings, B; Saris, WHM; Kies, AK; Kuipers, H; Van Loon, LJC (2007) Coingestion of carbohydrate with protein does not further augment postexercise muscle protein synthesis. AMERICAN JOURNAL OF PHYSIOLOGY-ENDOCRINOLOGY AND METABOLISM, 293:3, E833-E842. |
| 1. Betts, J; Williams, C; Duffy, K; Gunner, F (2007) The influence of carbohydrate and protein ingestion during recovery from prolonged exercise on subsequent endurance performance. JOURNAL OF SPORTS SCIENCES, 25:13, 1449-1460. |
| 1. Rowlands, DS; Thorp, RM; Rossler, K; Graham, DF; Rockell, MJ (2007) Effect of protein-rich feeding on recovery after intense exercise. INTERNATIONAL JOURNAL OF SPORT NUTRITION AND EXERCISE METABOLISM, 17:6, 521-543. |
| 1. Beelen, M; Koopman, R; Gijsen, AP; Vandereyt, H; Kies, AK; Kuipers, H; Saris, WHM; van Loon, LJC (2008) Protein coingestion stimulates muscle protein synthesis during resistance-type exercise. AMERICAN JOURNAL OF PHYSIOLOGY-ENDOCRINOLOGY AND METABOLISM, 295:1, E70-E77. |
| 1. Howarth, KR; Moreau, NA; Phillips, SM; Gibala, MJ (2009) Coingestion of protein with carbohydrate during recovery from endurance exercise stimulates skeletal muscle protein synthesis in humans. JOURNAL OF APPLIED PHYSIOLOGY, 106:4, 1394-1402. |
| 1. Kerksick, C; Harvey, T; Stout, J; Campbell, B; Wilborn, C; Kreider, R; Kalman, D; Ziegenfuss, T; Lopez, H; Landis, J; Ivy, JL; Antonio, J (2008) International Society of Sports Nutrition position stand: Nutrient timing. JOURNAL OF THE INTERNATIONAL SOCIETY OF SPORTS NUTRITION, 5:, -. |
| 1. Cermak, NM; Solheim, AS; Gardner, MS; Tarnopolsky, MA; Gibala, MJ (2009) Muscle Metabolism during Exercise with Carbohydrate or Protein-Carbohydrate Ingestion. MEDICINE AND SCIENCE IN SPORTS AND EXERCISE, 41:12, 2158-2164. |
| 1. Triplett, D; Doyle, JA; Rupp, JC; Benardot, D (2010) An Isocaloric Glucose-Fructose Beverage's Effect on Simulated 100-km Cycling Performance Compared With a Glucose-Only Beverage. INTERNATIONAL JOURNAL OF SPORT NUTRITION AND EXERCISE METABOLISM, 20:2, 122-131. |
| 1. Breen, L; Tipton, KD; Jeukendrup, AE (2010) No Effect of Carbohydrate-Protein on Cycling Performance and Indices of Recovery. MEDICINE AND SCIENCE IN SPORTS AND EXERCISE, 42:6, 1140-1148. |
| 1. Beelen, M; Burke, LM; Gibala, MJ; van Loon, LJC (2010) Nutritional Strategies to Promote Postexercise Recovery. INTERNATIONAL JOURNAL OF SPORT NUTRITION AND EXERCISE METABOLISM, 20:6, 515-532. |
| 1. Pfeiffer, B; Stellingwerff, T; Zaltas, E; Hodgson, AB; Jeukendrup, AE (2011) Carbohydrate Oxidation from a Drink during Running Compared with Cycling Exercise. MEDICINE AND SCIENCE IN SPORTS AND EXERCISE, 43:2, 327-334. |
| 1. Beelen, M; Van Kranenburg, J; Senden, JM; Kuipers, H; Van Loon, LJC (2012) Impact of Caffeine and Protein on Postexercise Muscle Glycogen Synthesis. MEDICINE AND SCIENCE IN SPORTS AND EXERCISE, 44:4, 692-700. |
| 1. Highton, J; Twist, C; Lamb, K; Nicholas, C (2013) Carbohydrate-protein coingestion improves multiple-sprint running performance. JOURNAL OF SPORTS SCIENCES, 31:4, 361-369. |
| 1. Cermak, NM; Loon, L (2013) The Use of Carbohydrates During Exercise as an Ergogenic Aid. SPORTS MEDICINE, 43:11, 1139-1155. |
| 1. Alghannam, AF; Tsintzas, K; Thompson, D; Bilzon, J; Betts, JA (2014) Exploring mechanisms of fatigue during repeated exercise and the dose dependent effects of carbohydrate and protein ingestion: study protocol for a randomised controlled trial. TRIALS, 15:, -. |
| 1. Kerksick, CM; Arent, S; Schoenfeld, BJ; Stout, JR; Campbell, B; Wilborn, CD; Taylor, L; Kalman, D; Smith-Ryan, AE; Kreider, RB; Willoughby, D; Arciero, PJ; VanDusseldorp, TA; Ormsbee, MJ; Wildman, R; Greenwood, M; Ziegenfuss, TN; Aragon, AA; Antonio, J (2017) International society of sports nutrition position stand: nutrient timing. JOURNAL OF THE INTERNATIONAL SOCIETY OF SPORTS NUTRITION, 14:, -. |
| 1. Gejl, KD; Thams, LB; Hansen, M; Rokkedal-Lausch, T; Plomgaard, P; Nybo, L; Larsen, FJ; Cardinale, DA; Jensen, K; Holmberg, HC; Vissing, K; Krtenblad, N (2017) No Superior Adaptations to Carbohydrate Periodization in Elite Endurance Athletes. MEDICINE AND SCIENCE IN SPORTS AND EXERCISE, 49:12, 2486-2497. |
| 1. McCartney, D; Desbrow, B; Irwin, C (2018) Post-exercise Ingestion of Carbohydrate, Protein and Water: A Systematic Review and Meta-analysis for Effects on Subsequent Athletic Performance. SPORTS MEDICINE, 48:2, 379-408. |
| 1. Andersson-Hall, U; Pettersson, S; Edin, F; Pedersen, A; Malmodin, D; Madsen, K (2018) Metabolism and Whole-Body Fat Oxidation Following Postexercise Carbohydrate or Protein Intake. INTERNATIONAL JOURNAL OF SPORT NUTRITION AND EXERCISE METABOLISM, 28:1, 37-45. |
| 1. Gejl, KD; Vissing, K; Hansen, M; Thams, L; Rokkedal-Lausch, T; Plomgaard, P; Lundby, AKM; Nybo, L; Jensen, K; Holmberg, HC; Ortenblad, N (2018) Changes in metabolism but not myocellular signaling by training with CHO-restriction in endurance athletes. PHYSIOLOGICAL REPORTS, 6:17, -. |
| 1. Sollie, O; Jeppesen, PB; Tangen, DS; Jerneren, F; Nellemann, B; Valsdottir, D; Madsen, K; Turner, C; Refsum, H; Skalhegg, BS; Ivy, JL; Jensen, J (2018) Protein intake in the early recovery period after exhaustive exercise improves performance the following day. JOURNAL OF APPLIED PHYSIOLOGY, 125:6, 1731-1742. |
| 1. Glace, BW; Kremenic, IJ; McHugh, MP (2019) Effect of carbohydrate beverage ingestion on central versus peripheral fatigue: a placebo-controlled, randomized trial in cyclists. APPLIED PHYSIOLOGY NUTRITION AND METABOLISM, 44:2, 139-147. |
| 1. WILLIAMS, C; BREWER, J; WALKER, M (1992) THE EFFECT OF A HIGH-CARBOHYDRATE DIET ON RUNNING PERFORMANCE DURING A 30-KM TREADMILL TIME TRIAL. EUROPEAN JOURNAL OF APPLIED PHYSIOLOGY AND OCCUPATIONAL PHYSIOLOGY, 65:1, 18-24. |
| 1. TSINTZAS, OK; WILLIAMS, C; BOOBIS, L; GREENHAFF, P (1995) CARBOHYDRATE INGESTION AND GLYCOGEN UTILIZATION IN DIFFERENT MUSCLE-FIBER TYPES IN MAN. JOURNAL OF PHYSIOLOGY-LONDON, 489:1, 243-250. |

**3) Muscle physiology: alkalosis and acidosis**


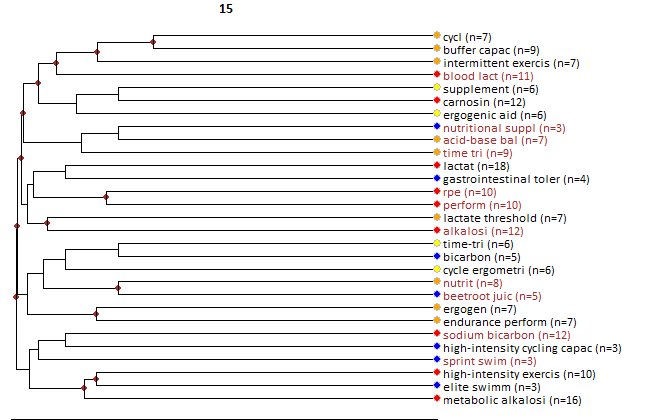


Core documents

| 1. Artioli, GG; Gualano, B; Coelho, DF; Benatti, FB; Galley, AW; Lancha, AH (2007) Does sodium-bicarbonate ingestion improve simulated judo performance?. INTERNATIONAL JOURNAL OF SPORT NUTRITION AND EXERCISE METABOLISM, 17:2, 206-217. |
| --- |
| 1. Carr, AJ; Gore, CJ; Dawson, B (2011) Induced Alkalosis and Caffeine Supplementation: Effects on 2,000-m Rowing Performance. INTERNATIONAL JOURNAL OF SPORT NUTRITION AND EXERCISE METABOLISM, 21:5, 357-364. |
| 1. Carr, AJ; Hopkins, WG; Gore, CJ (2011) Effects of Acute Alkalosis and Acidosis on Performance A Meta-Analysis. SPORTS MEDICINE, 41:10, 801-814. |
| 1. Bellinger, PM; Howe, ST; Shing, CM; Fell, JW (2012) Effect of Combined beta-Alanine and Sodium Bicarbonate Supplementation on Cycling Performance. MEDICINE AND SCIENCE IN SPORTS AND EXERCISE, 44:8, 1545-1551. |
| 1. Hobson, RM; Harris, RC; Martin, D; Smith, P; Macklin, B; Gualano, B; Sale, C (2013) Effect of Beta-Alanine With and Without Sodium Bicarbonate on 2,000-m Rowing Performance. INTERNATIONAL JOURNAL OF SPORT NUTRITION AND EXERCISE METABOLISM, 23:5, 480-487. |
| 1. Howe, ST; Bellinger, PM; Driller, MW; Shing, CM; Fell, JW (2013) The Effect of Beta-Alanine Supplementation on Isokinetic Force and Cycling Performance in Highly Trained Cyclists. INTERNATIONAL JOURNAL OF SPORT NUTRITION AND EXERCISE METABOLISM, 23:6, 562-570. |
| 1. Olek, RA; Kujach, S; Wnuk, D; Laskowski, R (2014) Single Sodium Pyruvate Ingestion Modifies Blood Acid-Base Status and Post-Exercise Lactate Concentration in Humans. NUTRIENTS, 6:5, 1981-1992. |
| 1. Gough, LA; Deb, SK; Sparks, SA; McNaughton, LR (2018) Sodium bicarbonate improves 4 km time trial cycling performance when individualised to time to peak blood bicarbonate in trained male cyclists. JOURNAL OF SPORTS SCIENCES, 36:15, 1705-1712. |
| 1. Delextrat, A; Mackessy, S; Arceo-Rendon, L; Scanlan, A; Ramsbottom, R; Calleja-Gonzalez, J (2018) Effects of Three-Day Serial Sodium Bicarbonate Loading on Performance and Physiological Parameters During a Simulated Basketball Test in Female University Players. INTERNATIONAL JOURNAL OF SPORT NUTRITION AND EXERCISE METABOLISM, 28:5, 547-552. |
| 1. Chycki, J; Golas, A; Halz, M; Maszczyk, A; Toborek, M; Zajac, A (2018) Chronic Ingestion of Sodium and Potassium Bicarbonate, with Potassium, Magnesium and Calcium Citrate Improves Anaerobic Performance in Elite Soccer Players. NUTRIENTS, 10:11, -. |
| 1. Ball, D; Maughan, RJ (1997) The effect of sodium citrate ingestion on the metabolic response to intense exercise following diet manipulation in man. EXPERIMENTAL PHYSIOLOGY, 82:6, 1041-1056. |

**4) Muscle power enhancement and dietary supplementation**


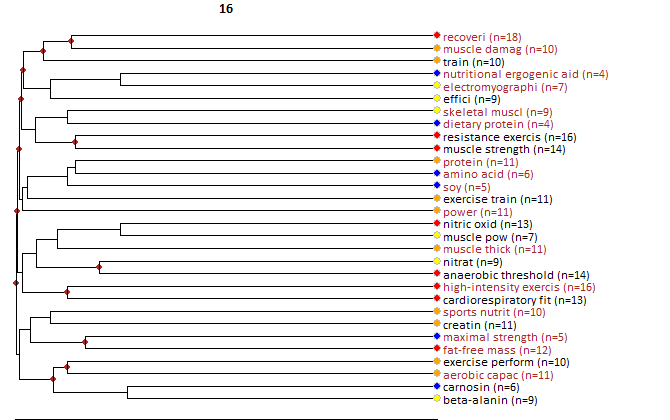


Core documents

| 1. Vandebuerie, F; Vanden Eynde, B; Vandenberghe, K; Hespel, P (1998) Effect of creatine loading on endurance capacity and sprint power in cyclists. INTERNATIONAL JOURNAL OF SPORTS MEDICINE, 19:7, 490-495. |
| --- |
| 1. Izquierdo, M; Ibanez, J; Gonzalez-Badillo, JJ; Gorostiaga, EM (2002) Effects of creatine supplementation on muscle power, endurance, and sprint performance. MEDICINE AND SCIENCE IN SPORTS AND EXERCISE, 34:2, 332-343. |
| 1. Rankin, JW; Goldman, LP; Puglisi, MJ; Nickols-Richardson, SM; Earthman, CP; Gwazdauskas, FC (2004) Effect of post-exercise supplement consumption on adaptations to resistance training. JOURNAL OF THE AMERICAN COLLEGE OF NUTRITION, 23:4, 322-330. |
| 1. Twist, C; Eston, R (2005) The effects of exercise-induced muscle damage on maximal intensity intermittent exercise performance. EUROPEAN JOURNAL OF APPLIED PHYSIOLOGY, 94:43591, 652-658. |
| 1. Kerksick, CM; Rasmussen, CJ; Lancaster, SL; Magu, B; Smith, P; Melton, C; Greenwood, M; Almada, AL; Earnest, CP; Kreider, RB (2006) The effects of protein and amino acid supplementation on performance and training adaptations during ten weeks of resistance training. JOURNAL OF STRENGTH AND CONDITIONING RESEARCH, 20:3, 643-653. |
| 1. Hoffman, J; Ratamess, N; Kang, J; Mangine, G; Faigenbaum, A; Stout, J (2006) Effect of creatine and beta-alanine supplementation on performance and endocrine responses in strength/power athletes. INTERNATIONAL JOURNAL OF SPORT NUTRITION AND EXERCISE METABOLISM, 16:4, 430-446. |
| 1. Smith, AE; Walter, AA; Graef, JL; Kendall, KL; Moon, JR; Lockwood, CM; Fukuda, DH; Beck, TW; Cramer, JT; Stout, JR (2009) Effects of beta-alanine supplementation and high-intensity interval training on endurance performance and body composition in men; a double-blind trial. JOURNAL OF THE INTERNATIONAL SOCIETY OF SPORTS NUTRITION, 6:, -. |
| 1. Mettler, S; Mitchell, N; Tipton, KD (2010) Increased Protein Intake Reduces Lean Body Mass Loss during Weight Loss in Athletes. MEDICINE AND SCIENCE IN SPORTS AND EXERCISE, 42:2, 326-337. |
| 1. Bemben, MG; Witten, MS; Carter, JM; Eliot, KA; Knehans, AW; Bemben, DA (2010) THE EFFECTS OF SUPPLEMENTATION WITH CREATINE AND PROTEIN ON MUSCLE STRENGTH FOLLOWING A TRADITIONAL RESISTANCE TRAINING PROGRAM IN MIDDLE-AGED AND OLDER MEN. JOURNAL OF NUTRITION HEALTH & AGING, 14:2, 155-159. |
| 1. Walter, AA; Smith, AE; Kendall, KL; Stout, JR; Cramer, JT (2010) SIX WEEKS OF HIGH-INTENSITY INTERVAL TRAINING WITH AND WITHOUT beta-ALANINE SUPPLEMENTATION FOR IMPROVING CARDIOVASCULAR FITNESS IN WOMEN. JOURNAL OF STRENGTH AND CONDITIONING RESEARCH, 24:5, 1199-1207. |
| 1. Hazell, TJ; MacPherson, REK; Gravelle, BMR; Lemon, PWR (2010) 10 or 30-s sprint interval training bouts enhance both aerobic and anaerobic performance. EUROPEAN JOURNAL OF APPLIED PHYSIOLOGY, 110:1, 153-160. |
| 1. Hickner, RC; Dyck, DJ; Sklar, J; Hatley, H; Byrd, P (2010) Effect of 28 days of creatine ingestion on muscle metabolism and performance of a simulated cycling road race. JOURNAL OF THE INTERNATIONAL SOCIETY OF SPORTS NUTRITION, 7:, -. |
| 1. Ormsbee, MJ; Mandler, WK; Thomas, DD; Ward, EG; Kinsey, AW; Simonavice, E; Panton, LB; Kim, JS (2012) The effects of six weeks of supplementation with multi-ingredient performance supplements and resistance training on anabolic hormones, body composition, strength, and power in resistance-trained men. JOURNAL OF THE INTERNATIONAL SOCIETY OF SPORTS NUTRITION, 9:, -. |
| 1. Veliz, RR; Requena, B; Suarez-Arrones, L; Newton, RU; de Villarreal, ES (2014) Effects of 18-Week In-Season Heavy-Resistance and Power Training on Throwing Velocity, Strength, Jumping, and Maximal Sprint Swim Performance of Elite Male Water Polo Players. JOURNAL OF STRENGTH AND CONDITIONING RESEARCH, 28:4, 1007-1014. |
| 1. Outlaw, JJ; Wilborn, CD; Smith-Ryan, AE; Hayward, SE; Urbina, SL; Taylor, LW; Foster, CA (2014) Acute effects of a commercially-available pre-workout supplement on markers of training: a double-blind study. JOURNAL OF THE INTERNATIONAL SOCIETY OF SPORTS NUTRITION, 11:, -. |
| 1. Negro, M; Vandoni, M; Ottobrini, S; Codrons, E; Correale, L; Buonocore, D; Marzatico, F (2014) Protein Supplementation with Low Fat Meat after Resistance Training: Effects on Body Composition and Strength. NUTRIENTS, 6:8, 3040-3049. |
| 1. Hazell, TJ; Hamilton, CD; Olver, TD; Lemon, PWR (2014) Running sprint interval training induces fat loss in women. APPLIED PHYSIOLOGY NUTRITION AND METABOLISM, 39:8, 944-950. |
| 1. Kresta, JY; Oliver, JM; Jagim, AR; Fluckey, J; Riechman, S; Kelly, K; Meininger, C; Mertens-Talcott, SU; Rasmussen, C; Kreider, RB (2014) Effects of 28 days of beta-alanine and creatine supplementation on muscle carnosine, body composition and exercise performance in recreationally active females. JOURNAL OF THE INTERNATIONAL SOCIETY OF SPORTS NUTRITION, 11:, -. |
| 1. Cochran, AJR; Percival, ME; Thompson, S; Gillen, JB; MacInnis, MJ; Potter, MA; Tamopolsky, MA; Gibala, MJ (2015) beta-Alanine Supplementation Does Not Augment the Skeletal Muscle Adaptive Response to 6 Weeks of Sprint Interval Training. INTERNATIONAL JOURNAL OF SPORT NUTRITION AND EXERCISE METABOLISM, 25:6, 541-549. |
| 1. Sheykhlouvand, M; Khalili, E; Agha-Alinejad, H; Gharaat, M (2016) HORMONAL AND PHYSIOLOGICAL ADAPTATIONS TO HIGH-INTENSITY INTERVAL TRAINING IN PROFESSIONAL MALE CANOE POLO ATHLETES. JOURNAL OF STRENGTH AND CONDITIONING RESEARCH, 30:3, 859-866. |
| 1. Bellinger, PM; Minahan, CL (2016) Additive Benefits of beta-Alanine Supplementation and Sprint-Interval Training. MEDICINE AND SCIENCE IN SPORTS AND EXERCISE, 48:12, 2417-2425. |
| 1. Bone, JL; Ross, ML; Tomcik, KA; Jeacocke, NA; Hopkins, WG; Burke, LM (2017) Manipulation of Muscle Creatine and Glycogen Changes Dual X-ray Absorptiometry Estimates of Body Composition. MEDICINE AND SCIENCE IN SPORTS AND EXERCISE, 49:5, 1029-1035. |
| 1. Cameron, M; Camic, CL; Doberstein, S; Erickson, JL; Jagim, AR (2018) The acute effects of a multi-ingredient pre-workout supplement on resting energy expenditure and exercise performance in recreationally active females. JOURNAL OF THE INTERNATIONAL SOCIETY OF SPORTS NUTRITION, 15:, -. |
| 1. Olek, RA; Kujach, S; Ziemann, E; Ziolkowski, W; Waz, P; Laskowski, R (2018) Adaptive Changes After 2 Weeks of 10-s Sprint Interval Training With Various Recovery Times. FRONTIERS IN PHYSIOLOGY, 9:, -. |
| 1. Patrizio, F; Ditroilo, M; Felici, F; Duranti, G; De Vito, G; Sabatini, S; Sacchetti, M; Bazzucchi, I (2018) The acute effect of Quercetin on muscle performance following a single resistance training session. EUROPEAN JOURNAL OF APPLIED PHYSIOLOGY, 118:5, 1021-1031. |
| 1. Santana, JO; de Freitas, MC; dos Santos, DM; Rossi, FE; Lira, FS; Rosa-Neto, JC; Caperuto, EC (2018) Beta-Alanine Supplementation Improved 10-km Running Time Trial in Physically Active Adults. FRONTIERS IN PHYSIOLOGY, 9:, -. |
| 1. Suarez-Arrones, L; de Villarreal, ES; Nunez, FJ; Di Salvo, V; Petri, C; Buccolini, A; Maldonado, RA; Torreno, N; Mendez-Villanueva, A (2018) In-season eccentric-overload training in elite soccer players: Effects on body composition, strength and sprint performance. PLOS ONE, 13:10, -. |

**5) Fluid balance and hydration**


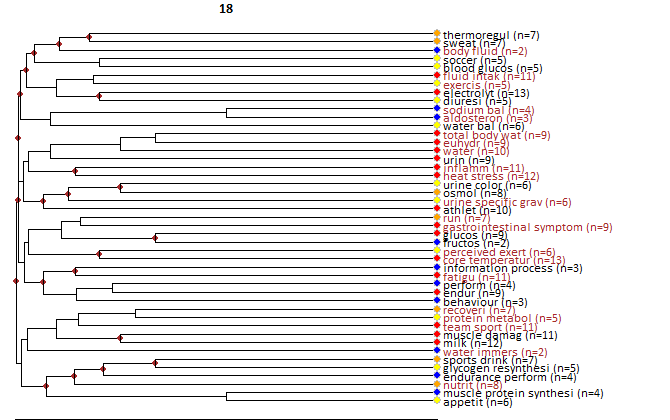


Core documents

| 1. Shirreffs, SM; Armstrong, LE; Cheuvront, SN (2004) Fluid and electrolyte needs for preparation and recovery from training and competition. JOURNAL OF SPORTS SCIENCES, 22:1, 57-63. |
| --- |
| 1. Maughan, RJ (1991) Fluid and electrolyte loss and replacement in exercise. JOURNAL OF SPORTS SCIENCES, 9:, 117-142. |
| 1. Logan-Sprenger, HM; Heigenhauser, GJF; Jones, GL; Spriet, LL (2015) The effect of dehydration on muscle metabolism and time trial performance during prolonged cycling in males. PHYSIOLOGICAL REPORTS, 3:8, -. |
| 1. Maughan, RJ; Merson, SJ; Broad, NP; Shirreffs, SM (2004) Fluid and electrolyte intake and loss in elite soccer players during training. INTERNATIONAL JOURNAL OF SPORT NUTRITION AND EXERCISE METABOLISM, 14:3, 333-346. |
| 1. Watson, P; Black, KE; Clark, SC; Maughan, RJ (2006) Exercise in the heat: Effect of fluid ingestion on blood-brain barrier permeability. MEDICINE AND SCIENCE IN SPORTS AND EXERCISE, 38:12, 2118-2124. |
| 1. Shirreffs, SM; Watson, P; Maughan, RJ (2007) Milk as an effective post-exercise rehydration drink. BRITISH JOURNAL OF NUTRITION, 98:1, 173-180. |
| 1. Evans, GH; Shirreffs, SM; Maughan, RJ (2009) Postexercise rehydration in man: The effects of osmolality and carbohydrate content of ingested drinks. NUTRITION, 25:9, 905-913. |
| 1. Maughan, RJ; Dargavel, LA; Hares, R; Shirreffs, SM (2009) Water and Salt Balance of Well-Trained Swimmers in Training. INTERNATIONAL JOURNAL OF SPORT NUTRITION AND EXERCISE METABOLISM, 19:6, 598-606. |
| 1. James, LJ; Clayton, D; Evans, GH (2011) Effect of milk protein addition to a carbohydrate-electrolyte rehydration solution ingested after exercise in the heat. BRITISH JOURNAL OF NUTRITION, 105:3, 393-399. |
| 1. de Oliveira, EP; Burini, RC (2011) Food-dependent, exercise-induced gastrointestinal distress. JOURNAL OF THE INTERNATIONAL SOCIETY OF SPORTS NUTRITION, 8:, -. |
| 1. Beis, LY; Polyviou, T; Malkova, D; Pitsiladis, YP (2011) The effects of creatine and glycerol hyperhydration on running economy in well trained endurance runners. JOURNAL OF THE INTERNATIONAL SOCIETY OF SPORTS NUTRITION, 8:, -. |
| 1. Kalman, DS; Feldman, S; Krieger, DR; Bloomer, RJ (2012) Comparison of coconut water and a carbohydrate-electrolyte sport drink on measures of hydration and physical performance in exercise-trained men. JOURNAL OF THE INTERNATIONAL SOCIETY OF SPORTS NUTRITION, 9:, -. |
| 1. O'Neal, EK; Davis, BA; Thigpen, LK; Caufield, CR; Horton, AD; McIntosh, JR (2012) Runners Greatly Underestimate Sweat Losses Before and After a 1-hr Summer Run. INTERNATIONAL JOURNAL OF SPORT NUTRITION AND EXERCISE METABOLISM, 22:5, 353-362. |
| 1. O'Neal, EK; Canfield, CR; Lowe, JB; Stevenson, MC; Davis, BA; Thigpen, LK (2014) 24-h Fluid Kinetics and Perception of Sweat Losses Following a 1-h Run in a Temperate Environment. NUTRIENTS, 6:1, 37-49. |
| 1. Davis, BA; Thigpen, LK; Hornsby, JH; Green, JM; Coates, TE; O'Neal, EK (2014) Hydration kinetics and 10-km outdoor running performance following 75% versus 150% between bout fluid replacement. EUROPEAN JOURNAL OF SPORT SCIENCE, 14:7, 703-710. |
| 1. Jimenez-Pavon, D; Cervantes-Borunda, MS; Diaz, LE; Marcos, A; Castillo, MJ (2015) Effects of a moderate intake of beer on markers of hydration after exercise in the heat: a crossover study. JOURNAL OF THE INTERNATIONAL SOCIETY OF SPORTS NUTRITION, 12:, -. |
| 1. Morris, DM; Huot, JR; Jetton, AM; Collier, SR; Utter, AC (2015) Acute Sodium Ingestion Before Exercise Increases Voluntary Water Consumption Resulting in Preexercise Hyperhydration and Improvement in Exercise Performance in the Heat. INTERNATIONAL JOURNAL OF SPORT NUTRITION AND EXERCISE METABOLISM, 25:5, 456-462. |
| 1. Wilcoxson, MCS; Johnson, SL; Pribyslayska, V; Green, JM; O'Neal, EK (2017) Fluid Retention and Utility of Practical Hydration Markers to Detect Three Levels of Recovery Fluid Intake in Male Runners. INTERNATIONAL JOURNAL OF SPORT NUTRITION AND EXERCISE METABOLISM, 27:2, 178-185. |
| 1. Evans, GH; James, LJ; Shirreffs, SM; Maughan, RJ (2017) Optimizing the restoration and maintenance of fluid balance after exercise-induced dehydration. JOURNAL OF APPLIED PHYSIOLOGY, 122:4, 945-951. |
| 1. Evans, GH; Miller, J; Whiteley, S; James, LJ (2017) A Sodium Drink Enhances Fluid Retention During 3 Hours of Post-Exercise Recovery When Ingested With a Standard Meal. INTERNATIONAL JOURNAL OF SPORT NUTRITION AND EXERCISE METABOLISM, 27:4, 344-350. |
| 1. de Souza, RF; de Oliveira, LS; de Matos, DG; Moreira, OC; da Silva, TC; Chilibeck, P; Ferreira, AR; Zanona, AD; Aidar, FJ (2018) Is sodium a good hyperhydration strategy in 10k runners?. JOURNAL OF HUMAN SPORT AND EXERCISE, 13:4, 823-831. |
| 1. WALSH, RM; NOAKES, TD; HAWLEY, JA; DENNIS, SC (1994) IMPAIRED HIGH-INTENSITY CYCLING PERFORMANCE TIME AT LOW-LEVELS OF DEHYDRATION. INTERNATIONAL JOURNAL OF SPORTS MEDICINE, 15:7, 392-398. |
| 1. MAUGHAN, RJ; LEIPER, JB (1995) SODIUM-INTAKE AND POSTEXERCISE REHYDRATION IN MAN. EUROPEAN JOURNAL OF APPLIED PHYSIOLOGY AND OCCUPATIONAL PHYSIOLOGY, 71:4, 311-319. |
| 1. Maughan, RJ; Leiper, JB; Shirreffs, SM (1996) Restoration of fluid balance after exercise-induced dehydration: Effects of food and fluid intake. EUROPEAN JOURNAL OF APPLIED PHYSIOLOGY AND OCCUPATIONAL PHYSIOLOGY, 73:43528, 317-325. |
| 1. Shirreffs, SM; Taylor, AJ; Leiper, JB; Maughan, RJ (1996) Post-exercise rehydration in man: Effects of volume consumed and drink sodium content. MEDICINE AND SCIENCE IN SPORTS AND EXERCISE, 28:10, 1260-1271. |

**6) Dietary intake and nutrition knowledge**


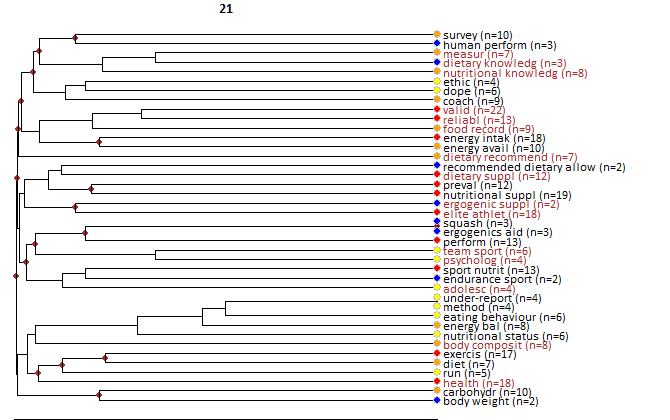


Core documents

| 1. Rockwell, MS; Nickols-Richardson, SM; Thye, FW (2001) Nutrition knowledge, opinions, and practices of coaches and athletic trainers at a Division I university. INTERNATIONAL JOURNAL OF SPORT NUTRITION AND EXERCISE METABOLISM, 11:2, 174-185. |
| --- |
| 1. Burns, RD; Schiller, MR; Merrick, MA; Wolf, KN (2004) Intercollegiate student athlete use of nutritional supplements and the role of athletic trainers and dietitians in nutrition counseling. JOURNAL OF THE AMERICAN DIETETIC ASSOCIATION, 104:2, 246-249. |
| 1. Nieper, A (2005) Nutritional supplement practices in UK junior national track and field athletes. BRITISH JOURNAL OF SPORTS MEDICINE, 39:9, 645-649. |
| 1. Jessri, M; Jessri, M; RashidKhani, B; Zinn, C (2010) Evaluation of Iranian College Athletes' Sport Nutrition Knowledge. INTERNATIONAL JOURNAL OF SPORT NUTRITION AND EXERCISE METABOLISM, 20:3, 257-263. |
| 1. Heaney, S; O'Connor, H; Michael, S; Gifford, J; Naughton, G (2011) Nutrition Knowledge in Athletes: A Systematic Review. INTERNATIONAL JOURNAL OF SPORT NUTRITION AND EXERCISE METABOLISM, 21:3, 248-261. |
| 1. Walsh, M; Cartwright, L; Corish, C; Sugrue, S; Wood-Martin, R (2011) The Body Composition, Nutritional Knowledge, Attitudes, Behaviors, and Future Education Needs of Senior Schoolboy Rugby Players in Ireland. INTERNATIONAL JOURNAL OF SPORT NUTRITION AND EXERCISE METABOLISM, 21:5, 365-376. |
| 1. Spronk, I; Kullen, C; Burdon, C; O'Connor, H (2014) Relationship between nutrition knowledge and dietary intake. BRITISH JOURNAL OF NUTRITION, 111:10, 1713-1726. |
| 1. Wiens, K; Erdman, KA; Stadnyk, M; Parnell, JA (2014) Dietary Supplement Usage, Motivation, and Education in Young Canadian Athletes. INTERNATIONAL JOURNAL OF SPORT NUTRITION AND EXERCISE METABOLISM, 24:6, 613-622. |
| 1. Alaunyte, I; Perry, JL; Aubrey, T (2015) Nutritional knowledge and eating habits of professional rugby league players: does knowledge translate into practice?. JOURNAL OF THE INTERNATIONAL SOCIETY OF SPORTS NUTRITION, 12:, -. |
| 1. Devlin, BL; Belski, R (2015) Exploring General and Sports Nutrition and Food Knowledge in Elite Male Australian Athletes. INTERNATIONAL JOURNAL OF SPORT NUTRITION AND EXERCISE METABOLISM, 25:3, 225-232. |
| 1. Spronk, I; Heaney, SE; Prvan, T; O'Connor, HT (2015) Relationship Between General Nutrition Knowledge and Dietary Quality in Elite Athletes. INTERNATIONAL JOURNAL OF SPORT NUTRITION AND EXERCISE METABOLISM, 25:3, 243-251. |
| 1. Couture, S; Lamarche, B; Morissette, E; Provencher, V; Valois, P; Goulet, C; Drapeau, V (2015) Evaluation of Sports Nutrition Knowledge and Recommendations Among High School Coaches. INTERNATIONAL JOURNAL OF SPORT NUTRITION AND EXERCISE METABOLISM, 25:4, 326-334. |
| 1. Andrews, MC; Itsiopoulos, C (2016) Room for Improvement in Nutrition Knowledge and Dietary Intake of Male Football (Soccer) Players in Australia. INTERNATIONAL JOURNAL OF SPORT NUTRITION AND EXERCISE METABOLISM, 26:1, 55-64. |
| 1. Tawfik, S; El Koofy, N; Moawad, EMI (2016) Patterns of Nutrition and Dietary Supplements Use in Young Egyptian Athletes: A Community-Based Cross-Sectional Survey. PLOS ONE, 11:8, -. |
| 1. Trakman, GL; Forsyth, A; Devlin, BL; Belski, R (2016) A Systematic Review of Athletes' and Coaches' Nutrition Knowledge and Reflections on the Quality of Current Nutrition Knowledge Measures. NUTRIENTS, 8:9, -. |
| 1. Kelly, VG; Leveritt, MD; Brennan, CT; Slater, GJ; Jenkins, DG (2017) Prevalence, knowledge and attitudes relating to beta-alanine use among professional footballers. JOURNAL OF SCIENCE AND MEDICINE IN SPORT, 20:1, 12-16. |
| 1. Abbey, EL; Wright, CJ; Kirkpatrick, CM (2017) Nutrition practices and knowledge among NCAA Division III football players. JOURNAL OF THE INTERNATIONAL SOCIETY OF SPORTS NUTRITION, 14:, -. |
| 1. Trakman, GL; Forsyth, A; Hoye, R; Belski, R (2017) The nutrition for sport knowledge questionnaire (NSKQ): development and validation using classical test theory and Rasch analysis. JOURNAL OF THE INTERNATIONAL SOCIETY OF SPORTS NUTRITION, 14:, -. |
| 1. Whitehouse, G; Lawlis, T (2017) Protein supplements and adolescent athletes: A pilot study investigating the risk knowledge, motivations and prevalence of use. NUTRITION & DIETETICS, 74:5, 509-515. |
| 1. Badau, D; Talaghir, LG; Rus, V; Badau, A (2018) THE IMPACT OF THE NEEDS AND ROLES OF NUTRITION COUNSELLING IN SPORT. HUMAN SPORT MEDICINE, 18:2, 88-96. |
| 1. Jenner, SL; Trakman, G; Coutts, A; Kempton, T; Ryan, S; Forsyth, A; Belski, R (2018) Dietary intake of professional Australian football athletes surrounding body composition assessment. JOURNAL OF THE INTERNATIONAL SOCIETY OF SPORTS NUTRITION, 15:, -. |
| 1. Heikkila, M; Valve, R; Lehtovirta, M; Fogelholm, M (2018) Nutrition Knowledge Among Young Finnish Endurance Athletes and Their Coaches. INTERNATIONAL JOURNAL OF SPORT NUTRITION AND EXERCISE METABOLISM, 28:5, 522-527. |
| 1. Partida, S; Marshall, A; Henry, R; Townsend, J; Toy, A (2018) Attitudes toward Nutrition and Dietary Habits and Effectiveness of Nutrition Education in Active Adolescents in a Private School Setting: A Pilot Study. NUTRIENTS, 10:9, -. |
| 1. Argolo, D; Borges, J; Cavalcante, A; Silva, G; Maia, S; Ramos, A; Oliveira, E; Nascimento, M (2018) Poor dietary intake and low nutritional knowledge in adolescent and adult competitive athletes: a warning to table tennis players. NUTRICION HOSPITALARIA, 35:5, 1124-1130. |
| 1. Blennerhassett, C; McNaughton, LR; Cronin, L; Sparks, SA (2019) Development and Implementation of a Nutrition Knowledge Questionnaire for Ultraendurance Athletes. INTERNATIONAL JOURNAL OF SPORT NUTRITION AND EXERCISE METABOLISM, 29:1, 39-45. |

**7) Assessment of energy intake of athletes**


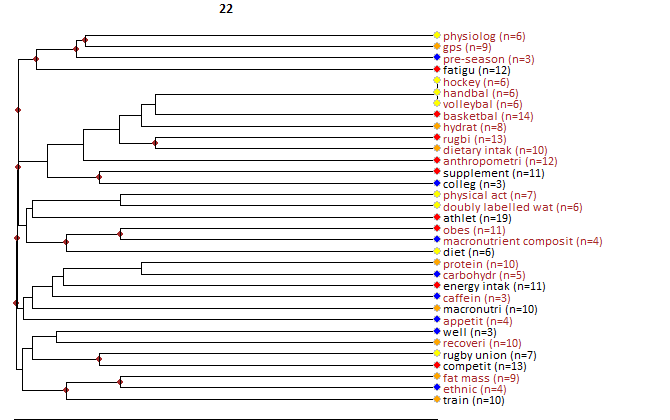


Core documents

| 1. Burke, LM; Slater, G; Broad, EM; Haukka, J; Modulon, S; Hopkins, WG (2003) Eating patterns and meal frequency of elite Australian athletes. INTERNATIONAL JOURNAL OF SPORT NUTRITION AND EXERCISE METABOLISM, 13:4, 521-538. |
| --- |
| 1. Ruiz, F; Irazusta, A; Gil, S; Irazusta, J; Casis, L; Gil, J (2005) Nutritional intake in soccer players of different ages. JOURNAL OF SPORTS SCIENCES, 23:3, 235-242. |
| 1. Lundy, B; O'Connor, H; Pelly, F; Caterson, I (2006) Anthropometric characteristics and competition dietary intakes of professional Rugby League players. INTERNATIONAL JOURNAL OF SPORT NUTRITION AND EXERCISE METABOLISM, 16:2, 199-213. |
| 1. Burke, LM; Loucks, AB; Broad, N (2006) Energy and carbohydrate for training and recovery. JOURNAL OF SPORTS SCIENCES, 24:7, 675-685. |
| 1. Dupont, G; Nedelec, M; McCall, A; McCormack, D; Berthoin, S; Wisloff, U (2010) Effect of 2 Soccer Matches in a Week on Physical Performance and Injury Rate. AMERICAN JOURNAL OF SPORTS MEDICINE, 38:9, 1752-1758. |
| 1. Russell, M; Pennock, A (2011) DIETARY ANALYSIS OF YOUNG PROFESSIONAL SOCCER PLAYERS FOR 1 WEEK DURING THE COMPETITIVE SEASON. JOURNAL OF STRENGTH AND CONDITIONING RESEARCH, 25:7, 1816-1823. |
| 1. Lago-Penas, C; Rey, E; Lago-Ballesteros, J; Casais, L; Dominguez, E (2011) THE INFLUENCE OF A CONGESTED CALENDAR ON PHYSICAL PERFORMANCE IN ELITE SOCCER. JOURNAL OF STRENGTH AND CONDITIONING RESEARCH, 25:8, 2111-2117. |
| 1. Skinner, AC; Hasty, SE; Turner, RW; Dreibelbis, M; Lohr, JA (2013) Is Bigger Really Better? Obesity Among High School Football Players, Player Position, and Team Success. CLINICAL PEDIATRICS, 52:10, 922-928. |
| 1. Bradley, WJ; Cavanagh, B; Douglas, W; Donovan, TF; Twist, C; Morton, JP; Close, GL (2015) Energy intake and expenditure assessed 'in-season' in an elite European rugby union squad. EUROPEAN JOURNAL OF SPORT SCIENCE, 15:6, 469-479. |
| 1. Briggs, MA; Cockburn, E; Rumbold, PLS; Rae, G; Stevenson, EJ; Russell, M (2015) Assessment of Energy Intake and Energy Expenditure of Male Adolescent Academy-Level Soccer Players during a Competitive Week. NUTRIENTS, 7:10, 8392-8401. |
| 1. Bilsborough, JC; Greenway, K; Livingston, S; Cordy, J; Coutts, AJ (2016) Changes in Anthropometry, Upper-Body Strength, and Nutrient Intake in Professional Australian Football Players During a Season. INTERNATIONAL JOURNAL OF SPORTS PHYSIOLOGY AND PERFORMANCE, 11:3, 290-300. |
| 1. Burrows, T; Harries, SK; Williams, RL; Lum, C; Callister, R (2016) The Diet Quality of Competitive Adolescent Male Rugby Union Players with Energy Balance Estimated Using Different Physical Activity Coefficients. NUTRIENTS, 8:9, -. |
| 1. Oliveira, CC; Ferreira, D; Caetano, C; Granja, D; Pinto, R; Mendes, B; Sousa, M (2017) Nutrition and Supplementation in Soccer. SPORTS, 5:2, -. |
| 1. Tsoufi, A; Maraki, MI; Dimitrakopoulos, L; Famisis, K; Grammatikopoulou, MG (2017) The effect of professional dietary counseling: elite basketball players eat healthier during competition days. JOURNAL OF SPORTS MEDICINE AND PHYSICAL FITNESS, 57:10, 1305-1310. |
| 1. Devlin, BL; Kingsley, M; Leveritt, MD; Belski, R (2017) SEASONAL CHANGES IN SOCCER PLAYERS' BODY COMPOSITION AND DIETARY INTAKE PRACTICES. JOURNAL OF STRENGTH AND CONDITIONING RESEARCH, 31:12, 3319-3326. |
| 1. Granja, DS; Cotovio, R; Pinto, R; Borrego, R; Mendes, L; Carolino, E; Macedo, P; Ferreira, D; Caetano, C; Mendes, B (2017) Evaluation of young elite soccer players food intake on match day and highest training load days. JOURNAL OF HUMAN SPORT AND EXERCISE, 12:4, 1238-1247. |
| 1. Black, KE; Black, AD; Baker, DF (2018) Macronutrient Intakes of Male Rugby Union Players: A Review. INTERNATIONAL JOURNAL OF SPORT NUTRITION AND EXERCISE METABOLISM, 28:6, 664-673. |
| 1. De Silva, V; Caine, M; Skinner, J; Dogan, S; Kondoz, A; Peter, T; Axtell, E; Birnie, M; Smith, B (2018) Player Tracking Data Analytics as a Tool for Physical Performance Management in Football: A Case Study from Chelsea Football Club Academy. SPORTS, 6:4, -. |
| 1. Maughan, RJ (1997) Energy and macronutrient intakes of professional football (soccer) players. BRITISH JOURNAL OF SPORTS MEDICINE, 31:1, 45-47. |

**8) Bone health, female athlete triad**


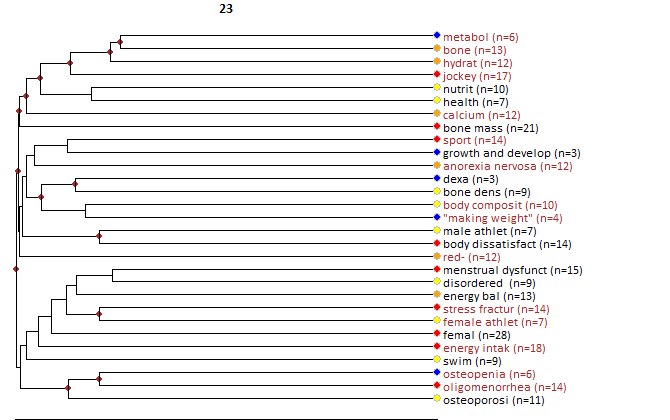


Core documents

| 1. Gibson, JH; Mitchell, A; Harries, MG; Reeve, J (2004) Nutritional and exercise-related determinants of bone density in elite female runners. OSTEOPOROSIS INTERNATIONAL, 15:8, 611-618. |
| --- |
| 1. Rouveix, M; Bouget, M; Pannafieux, C; Champely, S; Filaire, E (2007) Eating attitudes, body esteem, perfectionism and anxiety of judo athletes and nonathletes. INTERNATIONAL JOURNAL OF SPORTS MEDICINE, 28:4, 340-345. |
| 1. Loucks, AB (2007) Low energy availability in the marathon and other endurance sports. SPORTS MEDICINE, 37:43560, 348-352. |
| 1. Barrack, MT; Rauh, MJ; Nichols, JF (2008) Prevalence of and Traits Associated with Low BMD among Female Adolescent Runners. MEDICINE AND SCIENCE IN SPORTS AND EXERCISE, 40:12, 2015-2021. |
| 1. Hoch, AZ; Pajewski, NM; Moraski, L; Carrrera, GF; Wilson, CR; Hoffmann, RG; Schimke, JE; Gutterman, DD (2009) Prevalence of the Female Athlete Triad in High School Athletes and Sedentary Students. CLINICAL JOURNAL OF SPORT MEDICINE, 19:5, 421-428. |
| 1. Rauh, MJ; Nichols, JF; Barrack, MT (2010) Relationships Among Injury and Disordered Eating, Menstrual Dysfunction, and Low Bone Mineral Density in High School Athletes: A Prospective Study. JOURNAL OF ATHLETIC TRAINING, 45:3, 243-252. |
| 1. Barrack, MT; Van Loan, MD; Rauh, MJ; Nichols, JF (2010) Physiologic and behavioral indicators of energy deficiency in female adolescent runners with elevated bone turnover. AMERICAN JOURNAL OF CLINICAL NUTRITION, 92:3, 652-659. |
| 1. Hoch, AZ; Papanek, P; Szabo, A; Widlansky, ME; Schimke, JE; Gutterman, DD (2011) Association Between the Female Athlete Triad and Endothelial Dysfunction in Dancers. CLINICAL JOURNAL OF SPORT MEDICINE, 21:2, 119-125. |
| 1. Ducher, G; Turner, AI; Kukuljan, S; Pantano, KJ; Carlson, JL; Williams, NL; De Souza, MJ (2011) Obstacles in the Optimization of Bone Health Outcomes in the Female Athlete Triad. SPORTS MEDICINE, 41:7, 587-607. |
| 1. Miller, SM; Kukuljan, S; Turner, AI; van der Pligt, P; Ducher, G (2012) Energy Deficiency, Menstrual Disturbances, and Low Bone Mass: What Do Exercising Australian Women Know About the Female Athlete Triad?. INTERNATIONAL JOURNAL OF SPORT NUTRITION AND EXERCISE METABOLISM, 22:2, 131-138. |
| 1. Torstveit, MK; Sundgot-Borgen, J (2012) Are Under- and Overweight Female Elite Athletes Thin and Fat? A Controlled Study. MEDICINE AND SCIENCE IN SPORTS AND EXERCISE, 44:5, 949-957. |
| 1. Scofield, KL; Hecht, S (2012) Bone Health in Endurance Athletes: Runners, Cyclists, and Swimmers. CURRENT SPORTS MEDICINE REPORTS, 11:6, 328-334. |
| 1. Barrack, MT; Gibbs, JC; De Souza, MJ; Williams, NI; Nichols, JF; Rauh, MJ; Nattiv, A (2014) Higher Incidence of Bone Stress Injuries With Increasing Female Athlete Triad-Related Risk Factors A Prospective Multisite Study of Exercising Girls and Women. AMERICAN JOURNAL OF SPORTS MEDICINE, 42:4, 949-958. |
| 1. Tenforde, AS; Fredericson, M; Sayres, LC; Cutti, P; Sainani, KL (2015) Identifying Sex-Specific Risk Factors for Low Bone Mineral Density in Adolescent Runners. AMERICAN JOURNAL OF SPORTS MEDICINE, 43:6, 1494-1504. |
| 1. Thralls, KJ; Nichols, JF; Barrack, MT; Kern, M; Rauh, MJ (2016) Body Mass-Related Predictors of the Female Athlete Triad Among Adolescent Athletes. INTERNATIONAL JOURNAL OF SPORT NUTRITION AND EXERCISE METABOLISM, 26:1, 17-25. |
| 1. Muia, EN; Wright, HH; Onywera, VO; Kuria, EN (2016) Adolescent elite Kenyan runners are at risk for energy deficiency, menstrual dysfunction and disordered eating. JOURNAL OF SPORTS SCIENCES, 34:7, 598-606. |
| 1. Tenforde, AS; Barrack, MT; Nattiv, A; Fredericson, M (2016) Parallels with the Female Athlete Triad in Male Athletes. SPORTS MEDICINE, 46:2, 171-182. |
| 1. Blauwet, CA; Brook, EM; Tenforde, AS; Broad, E; Hu, CH; Abdu-Glass, E; Matzkin, EG (2017) Low Energy Availability, Menstrual Dysfunction, and Low Bone Mineral Density in Individuals with a Disability: Implications for the Para Athlete Population. SPORTS MEDICINE, 47:9, 1697-1708. |
| 1. Southmayd, EA; Hellmers, AC; De Souza, MJ (2017) Food Versus Pharmacy: Assessment of Nutritional and Pharmacological Strategies to Improve Bone Health in Energy-Deficient Exercising Women. CURRENT OSTEOPOROSIS REPORTS, 15:5, 459-472. |
| 1. Ahmad, NS; Hamid, MSA; Cheong, JPG; Hamzah, SH (2018) Bone Mineral Density and Associated Risk Factors among Female Athletes: A Cross-Sectional Study. SAINS MALAYSIANA, 47:1, 123-129. |
| 1. Heikura, IA; Uusitalo, ALT; Stellingwerff, T; Bergland, D; Mero, AA; Burke, LM (2018) Low Energy Availability Is Difficult to Assess but Outcomes Have Large Impact on Bone Injury Rates in Elite Distance Athletes. INTERNATIONAL JOURNAL OF SPORT NUTRITION AND EXERCISE METABOLISM, 28:4, 403-411. |
| 1. Daily, JP; Stumbo, JR (2018) Female Athlete Triad. PRIMARY CARE, 45:4, 615-+. |

**9) Fluid intake strategy**


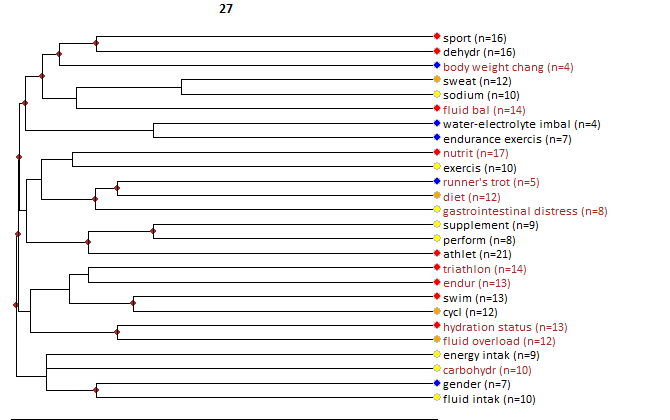


Core documents

| 1. Glace, B; Murphy, C; McHugh, M (2002) Food and fluid intake and disturbances in gastrointestinal and mental function during an ultramarathon. INTERNATIONAL JOURNAL OF SPORT NUTRITION AND EXERCISE METABOLISM, 12:4, 414-427. |
| --- |
| 1. Coyle, EF (2004) Fluid and fuel intake during exercise. JOURNAL OF SPORTS SCIENCES, 22:1, 39-55. |
| 1. Fudge, BW; Easton, C; Kingsmore, D; Kiplamai, FK; Onywera, VO; Westerterp, KR; Kayser, B; Noakes, TD; Pitsiladis, YP (2008) Elite Kenyan endurance runners are hydrated day-to-day with ad libitum fluid intake. MEDICINE AND SCIENCE IN SPORTS AND EXERCISE, 40:6, 1171-1179. |
| 1. Moran, ST; Dziedzic, CE; Cox, GR (2011) Feeding Strategies of a Female Athlete During an Ultraendurance Running Event. INTERNATIONAL JOURNAL OF SPORT NUTRITION AND EXERCISE METABOLISM, 21:4, 347-351. |
| 1. Garth, AK; Burke, LM (2013) What Do Athletes Drink During Competitive Sporting Activities?. SPORTS MEDICINE, 43:7, 539-564. |
| 1. Shaw, G; Koivisto, A; Gerrard, D; Burke, LM (2014) Nutrition Considerations for Open-Water Swimming. INTERNATIONAL JOURNAL OF SPORT NUTRITION AND EXERCISE METABOLISM, 24:4, 373-381. |
| 1. Barrero, A; Erola, P; Bescos, R (2015) Energy Balance of Triathletes during an Ultra-Endurance Event. NUTRIENTS, 7:1, 209-222. |
| 1. Buoite Stella, A; Francescato, MP; Sims, ST; Morison, SA (2017) Fluid intake behavior in athletes during typical training bouts. JOURNAL OF SPORTS MEDICINE AND PHYSICAL FITNESS, 57:11, 1504-1512. |
| 1. Wardenaar, FC; Hoogervorst, D; Versteegen, JJ; van der Burg, N; Lambrechtse, KJ; Bongers, CCWG (2018) Real-Time Observations of Food and Fluid Timing During a 120 km Ultramarathon. FRONTIERS IN NUTRITION, 5:, -. |
| 1. Nikolaidis, PT; Veniamakis, E; Rosemann, T; Knechtle, B (2018) Nutrition in Ultra-Endurance: State of the Art. NUTRIENTS, 10:12, -. |

**10) Weight management**


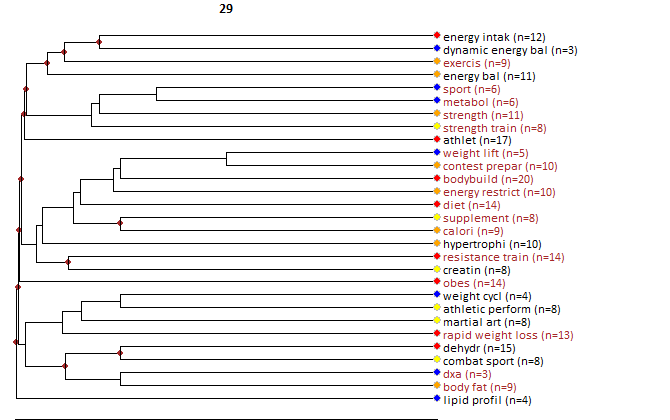


Core documents

| 1. Halton, TL; Hu, FB (2004) The effects of high protein diets on thermogenesis, satiety and weight loss: A critical review. JOURNAL OF THE AMERICAN COLLEGE OF NUTRITION, 23:5, 373-385. |
| --- |
| 1. Degoutte, F; Jouanel, P; Begue, RJ; Colombier, M; Lac, G; Pequignot, JM; Filaire, E (2006) Food restriction, performance, biochemical, psychological, and endocrine changes in judo athletes. INTERNATIONAL JOURNAL OF SPORTS MEDICINE, 27:1, 9-18. |
| 1. Stiegler, P; Cunliffe, A (2006) The role of diet and exercise for the maintenance of fat-free mass and resting metabolic rate during weight loss. SPORTS MEDICINE, 36:3, 239-262. |
| 1. Hagmar, M; Hirschberg, AL; Berglund, L; Berglund, B (2008) Special attention to the weight-control strategies employed by Olympic athletes striving for leanness is required. CLINICAL JOURNAL OF SPORT MEDICINE, 18:1, 5-9. |
| 1. Donnelly, JE; Blair, SN; Jakicic, JM; Manore, MM; Rankin, JW; Smith, BK (2009) Appropriate Physical Activity Intervention Strategies for Weight Loss and Prevention of Weight Regain for Adults. MEDICINE AND SCIENCE IN SPORTS AND EXERCISE, 41:2, 459-471. |
| 1. Artioli, GG; Iglesias, RT; Franchini, E; Gualano, B; Kashiwagura, DB; Solis, MY; Benatti, FB; Fuchs, M; Lancha, AH (2010) Rapid weight loss followed by recovery time does not affect judo-related performance. JOURNAL OF SPORTS SCIENCES, 28:1, 21-32. |
| 1. Morton, JP; Robertson, C; Sutton, L; MacLaren, DPM (2010) Making the Weight: A Case Study From Professional Boxing. INTERNATIONAL JOURNAL OF SPORT NUTRITION AND EXERCISE METABOLISM, 20:1, 80-85. |
| 1. Artioli, GG; Gualano, B; Franchini, E; Scagliusi, FB; Takesian, M; Fuchs, M; Lancha, AH (2010) Prevalence, Magnitude, and Methods of Rapid Weight Loss among Judo Competitors. MEDICINE AND SCIENCE IN SPORTS AND EXERCISE, 42:3, 436-442. |
| 1. Sagayama, H; Yoshimura, E; Yamada, Y; Ichikawa, M; Ebine, N; Higaki, Y; Kiyonaga, A; Tanaka, H (2014) Effects of rapid weight loss and regain on body composition and energy expenditure. APPLIED PHYSIOLOGY NUTRITION AND METABOLISM, 39:1, 21-27. |
| 1. Trexler, ET; Smith-Ryan, AE; Norton, LE (2014) Metabolic adaptation to weight loss: implications for the athlete. JOURNAL OF THE INTERNATIONAL SOCIETY OF SPORTS NUTRITION, 11:, -. |
| 1. Phillips, SM (2014) A Brief Review of Higher Dietary Protein Diets in Weight Loss: A Focus on Athletes. SPORTS MEDICINE, 44:, S149-S153. |
| 1. Durguerian, A; Bougard, C; Drogou, C; Sauvet, F; Chennaoui, M; Filaire, E (2016) Weight Loss, Performance and Psychological Related States in High-level Weightlifters. INTERNATIONAL JOURNAL OF SPORTS MEDICINE, 37:3, 230-238. |
| 1. Berkovich, BE; Eliakim, A; Nemet, D; Stark, AH; Sinai, T (2016) Rapid Weight Loss Among Adolescents Participating In Competitive Judo. INTERNATIONAL JOURNAL OF SPORT NUTRITION AND EXERCISE METABOLISM, 26:3, 276-284. |
| 1. Hulmi, JJ; Isola, V; Suonpaa, M; Jarvinen, NJ; Kokkonen, M; Wennerstrom, A; Nyman, K; Perola, M; Ahtiainen, JP; Hakkinen, K (2017) The Effects of Intensive Weight Reduction on Body Composition and Serum Hormones in Female Fitness Competitors. FRONTIERS IN PHYSIOLOGY, 7:, -. |
| 1. Matthews, JJ; Nicholas, C (2017) Extreme Rapid Weight Loss and Rapid Weight Gain Observed in UK Mixed Martial Arts Athletes Preparing for Competition. INTERNATIONAL JOURNAL OF SPORT NUTRITION AND EXERCISE METABOLISM, 27:2, 122-129. |
| 1. Hector, AJ; Phillips, SM (2018) Protein Recommendations for Weight Loss in Elite Athletes: A Focus on Body Composition and Performance. INTERNATIONAL JOURNAL OF SPORT NUTRITION AND EXERCISE METABOLISM, 28:2, 170-177. |
| 1. Sagayama, H; Shizuma, K; Toguchi, M; Mizuhara, H; Machida, Y; Yamada, Y; Ebine, N; Higaki, Y; Tanaka, H (2018) Effect of the Health Tourism weight loss programme on body composition and health outcomes in healthy and excess-weight adults. BRITISH JOURNAL OF NUTRITION, 119:10, 1133-1141. |
| 1. Reale, R; Slater, G; Burke, LM (2018) Weight management Practices of Australian Olympic Combat Sport Athletes. INTERNATIONAL JOURNAL OF SPORTS PHYSIOLOGY AND PERFORMANCE, 13:4, 459-466. |
| 1. WALBERGRANKIN, J; EDMONDS, CE; GWAZDAUSKAS, FC (1993) DIET AND WEIGHT CHANGES OF FEMALE BODYBUILDERS BEFORE AND AFTER COMPETITION. INTERNATIONAL JOURNAL OF SPORT NUTRITION, 3:1, 87-102. |

**11) Nutritional Strategies and human skeletal muscle**


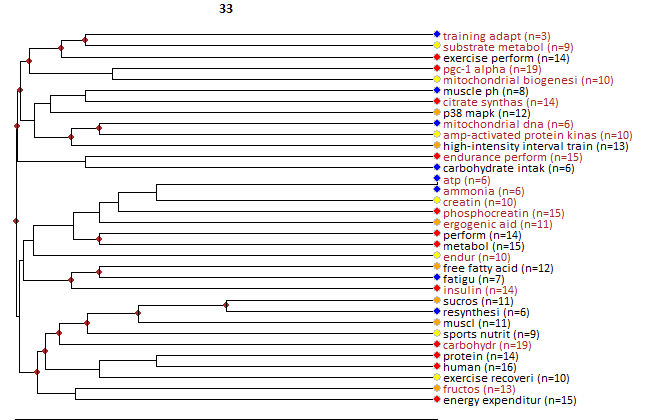


Core documents

| 1. Tsintzas, K; Williams, C (1998) Human muscle glycogen metabolism during exercise - Effect of carbohydrate supplementation. SPORTS MEDICINE, 25:1, 7-23. |
| --- |
| 1. Hargreaves, M; Hawley, JA; Jeukendrup, A (2004) Pre-exercise carbohydrate and fat ingestion: effects on metabolism and performance. JOURNAL OF SPORTS SCIENCES, 22:1, 31-38. |
| 1. Pilegaard, H; Osada, T; Andersen, LT; Helge, JW; Saltin, B; Neufer, PD (2005) Substrate availability and transcriptional regulation of metabolic genes in human skeletal muscle during recovery from exercise. METABOLISM-CLINICAL AND EXPERIMENTAL, 54:8, 1048-1055. |
| 1. Burgomaster, KA; Howarth, KR; Phillips, SM; Rakobowchuk, M; MacDonald, MJ; Mcgee, SL; Gibala, MJ (2008) Similar metabolic adaptations during exercise after low volume sprint interval and traditional endurance training in humans. JOURNAL OF PHYSIOLOGY-LONDON, 586:1, 151-160. |
| 1. Morton, JP; Croft, L; Bartlett, JD; MacLaren, DPM; Reilly, T; Evans, L; McArdle, A; Drust, B (2009) Reduced carbohydrate availability does not modulate training-induced heat shock protein adaptations but does upregulate oxidative enzyme activity in human skeletal muscle. JOURNAL OF APPLIED PHYSIOLOGY, 106:5, 1513-1521. |
| 1. Cochran, AJR; Little, JP; Tarnopolsky, MA; Gibala, MJ (2010) Carbohydrate feeding during recovery alters the skeletal muscle metabolic response to repeated sessions of high-intensity interval exercise in humans. JOURNAL OF APPLIED PHYSIOLOGY, 108:3, 628-636. |
| 1. Iaia, FM; Bangsbo, J (2010) Speed endurance training is a powerful stimulus for physiological adaptations and performance improvements of athletes. SCANDINAVIAN JOURNAL OF MEDICINE & SCIENCE IN SPORTS, 20:, 11-23. |
| 1. Betts, JA; Williams, C (2010) Short-Term Recovery from Prolonged Exercise Exploring the Potential for Protein Ingestion to Accentuate the Benefits of Carbohydrate Supplements. SPORTS MEDICINE, 40:11, 941-959. |
| 1. Psilander, N; Frank, P; Flockhart, M; Sahlin, K (2013) Exercise with low glycogen increases PGC-1 alpha gene expression in human skeletal muscle. EUROPEAN JOURNAL OF APPLIED PHYSIOLOGY, 113:4, 951-963. |
| 1. Gibala, MJ (2013) Nutritional Strategies to Support Adaptation to High-Intensity Interval Training in Team Sports. NUTRITIONAL COACHING STRATEGY TO MODULATE TRAINING EFFICIENCY, 75:, 41-49. |
| 1. Aoi, W; Naito, Y; Yoshikawa, T (2013) Role of oxidative stress in impaired insulin signaling associated with exercise-induced muscle damage. FREE RADICAL BIOLOGY AND MEDICINE, 65:, 1265-1272. |
| 1. Spriet, LL (2014) New Insights into the Interaction of Carbohydrate and Fat Metabolism During Exercise. SPORTS MEDICINE, 44:, 87-96. |
| 1. Hawley, JA; Morton, JP (2014) Ramping up the signal: promoting endurance training adaptation in skeletal muscle by nutritional manipulation. CLINICAL AND EXPERIMENTAL PHARMACOLOGY AND PHYSIOLOGY, 41:8, 608-613. |
| 1. Bartlett, JD; Hawley, JA; Morton, JP (2015) Carbohydrate availability and exercise training adaptation: Too much of a good thing?. EUROPEAN JOURNAL OF SPORT SCIENCE, 15:1, 3-12. |
| 1. Percival, ME; Martin, BJ; Gillen, JB; Skelly, LE; MacInnis, MJ; Green, AE; Tarnopolsky, MA; Gibala, MJ (2015) Sodium bicarbonate ingestion augments the increase in PGC-1 alpha mRNA expression during recovery from intense interval exercise in human skeletal muscle. JOURNAL OF APPLIED PHYSIOLOGY, 119:11, 1303-1312. |
| 1. Knuiman, P; Hopman, MTE; Mensink, M (2015) Glycogen availability and skeletal muscle adaptations with endurance and resistance exercise. NUTRITION & METABOLISM, 12:, -. |
| 1. Morville, T; Rosenkilde, M; Munch-Andersen, T; Andersen, PR; Groenbaek, KK; Helbo, S; Kristensen, M; Hansen, AV; Mattsson, N; Rasmusen, HK; Guadalupe-Grau, A; Fago, A; Hansen, CN; Twelkmeyer, B; Andersen, JL; Dela, F; Helge, JW (2017) Repeated Prolonged Exercise Decreases Maximal Fat Oxidation in Older Men. MEDICINE AND SCIENCE IN SPORTS AND EXERCISE, 49:2, 308-316. |
| 1. MacInnis, MJ; Zacharewicz, E; Martin, BJ; Haikalis, ME; Skelly, LE; Tarnopolsky, MA; Murphy, RM; Gibala, MJ (2017) Superior mitochondrial adaptations in human skeletal muscle after interval compared to continuous single-leg cycling matched for total work. JOURNAL OF PHYSIOLOGY-LONDON, 595:9, 2955-2968. |
| 1. Goncalves, NG; Cavaletti, SH; Pasqualucci, CA; Martins, MA; Lin, CJ (2017) Fructose ingestion impairs expression of genes involved in skeletal muscle's adaptive response to aerobic exercise. GENES AND NUTRITION, 12:, -. |
| 1. Knuiman, P; Hopman, MTE; Wouters, JA; Mensink, M (2018) Select Skeletal Muscle mRNAs Related to Exercise Adaptation Are Minimally Affected by Different Pre-exercise Meals that Differ in Macronutrient Profile. FRONTIERS IN PHYSIOLOGY, 9:, -. |
| 1. Hearris, MA; Hammond, KM; Fell, JM; Morton, JP (2018) Regulation of Muscle Glycogen Metabolism during Exercise: Implications for Endurance Performance and Training Adaptations. NUTRIENTS, 10:3, -. |
| 1. Knuiman, P; Hopman, MTE; Verbruggen, C; Mensink, M (2018) Protein and the Adaptive Response With Endurance Training: Wishful Thinking or a Competitive Edge?. FRONTIERS IN PHYSIOLOGY, 9:, -. |

**12) Dietary nitrate supplementation**


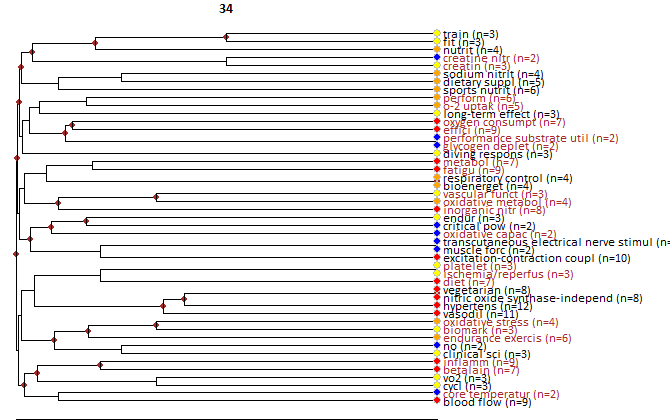


Core documents

| 1. Larsen, FJ; Weitzberg, E; Lundberg, JO; Ekblom, B (2007) Effects of dietary nitrate on oxygen cost during exercise. ACTA PHYSIOLOGICA, 191:1, 59-66. |
| --- |
| 1. Bailey, SJ; Winyard, P; Vanhatalo, A; Blackwell, JR; DiMenna, FJ; Wilkerson, DP; Tarr, J; Benjamin, N; Jones, AM (2009) Dietary nitrate supplementation reduces the O-2 cost of low-intensity exercise and enhances tolerance to high-intensity exercise in humans. JOURNAL OF APPLIED PHYSIOLOGY, 107:4, 1144-1155. |
| 1. Larsen, FJ; Weitzberg, E; Lundberg, JO; Ekblom, B (2010) Dietary nitrate reduces maximal oxygen consumption while maintaining work performance in maximal exercise. FREE RADICAL BIOLOGY AND MEDICINE, 48:2, 342-347. |
| 1. Kapil, V; Milsom, AB; Okorie, M; Maleki-Toyserkani, S; Akram, F; Rehman, F; Arghandawi, S; Pearl, V; Benjamin, N; Loukogeorgakis, S; MacAllister, R; Hobbs, AJ; Webb, AJ; Ahluwalia, A (2010) Inorganic Nitrate Supplementation Lowers Blood Pressure in Humans Role for Nitrite-Derived NO. HYPERTENSION, 56:2, 274-U174. |
| 1. Vanhatalo, A; Bailey, SJ; Blackwell, JR; DiMenna, FJ; Pavey, TG; Wilkerson, DP; Benjamin, N; Winyard, PG; Jones, AM (2010) Acute and chronic effects of dietary nitrate supplementation on blood pressure and the physiological responses to moderate-intensity and incremental exercise. AMERICAN JOURNAL OF PHYSIOLOGY-REGULATORY INTEGRATIVE AND COMPARATIVE PHYSIOLOGY, 299:4, R1121-R1131. |
| 1. Kenjale, AA; Ham, KL; Stabler, T; Robbins, JL; Johnson, JL; VanBruggen, M; Privette, G; Yim, E; Kraus, WE; Allen, JD (2011) Dietary nitrate supplementation enhances exercise performance in peripheral arterial disease. JOURNAL OF APPLIED PHYSIOLOGY, 110:6, 1582-1591. |
| 1. Bescos, R; Rodriguez, FA; Iglesias, X; Ferrer, MD; Iborra, E; Pons, A (2011) Acute Administration of Inorganic Nitrate Reduces (V) over dotO(2peak) in Endurance Athletes. MEDICINE AND SCIENCE IN SPORTS AND EXERCISE, 43:10, 1979-1986. |
| 1. Cermak, NM; Gibala, MJ; van Loon, LJC (2012) Nitrate Supplementation's Improvement of 10-km Time-Trial Performance in Trained Cyclists. INTERNATIONAL JOURNAL OF SPORT NUTRITION AND EXERCISE METABOLISM, 22:1, 64-71. |
| 1. Bond, H; Morton, L; Braakhuis, AJ (2012) Dietary Nitrate Supplementation Improves Rowing Performance in Well-Trained Rowers. INTERNATIONAL JOURNAL OF SPORT NUTRITION AND EXERCISE METABOLISM, 22:4, 251-256. |
| 1. Bescos, R; Ferrer-Roca, V; Galilea, PA; Roig, A; Drobnic, F; Sureda, A; Martorell, M; Cordova, A; Tur, JA; Pons, A (2012) Sodium Nitrate Supplementation Does Not Enhance Performance of Endurance Athletes. MEDICINE AND SCIENCE IN SPORTS AND EXERCISE, 44:12, 2400-2409. |
| 1. Kelly, J; Fulford, J; Vanhatalo, A; Blackwell, JR; French, O; Bailey, SJ; Gilchrist, M; Winyard, PG; Jones, AM (2013) Effects of short-term dietary nitrate supplementation on blood pressure, O-2 uptake kinetics, and muscle and cognitive function in older adults. AMERICAN JOURNAL OF PHYSIOLOGY-REGULATORY INTEGRATIVE AND COMPARATIVE PHYSIOLOGY, 304:2, R73-R83. |
| 1. Ferguson, SK; Hirai, DM; Copp, S; Holdsworth, CT; Allen, JD; Jones, AM; Musch, TI; Poole, DC (2013) Impact of dietary nitrate supplementation via beetroot juice on exercising muscle vascular control in rats. JOURNAL OF PHYSIOLOGY-LONDON, 591:2, 547-557. |
| 1. Jones, AM; Vanhatalo, A; Bailey, SJ (2013) Influence of Dietary Nitrate Supplementation on Exercise Tolerance and Performance. NUTRITIONAL COACHING STRATEGY TO MODULATE TRAINING EFFICIENCY, 75:, 27-40. |
| 1. Muggeridge, DJ; Howe, CCF; Spendiff, O; Pedlar, C; James, PE; Easton, C (2013) The Effects of a Single Dose of Concentrated Beetroot Juice on Performance in Trained Flatwater Kayakers. INTERNATIONAL JOURNAL OF SPORT NUTRITION AND EXERCISE METABOLISM, 23:5, 498-506. |
| 1. Hoon, MW; Johnson, NA; Chapman, PG; Burke, LM (2013) The Effect of Nitrate Supplementation on Exercise Performance in Healthy Individuals: A Systematic Review and Meta-Analysis. INTERNATIONAL JOURNAL OF SPORT NUTRITION AND EXERCISE METABOLISM, 23:5, 522-532. |
| 1. Jones, AM (2014) Dietary Nitrate Supplementation and Exercise Performance. SPORTS MEDICINE, 44:, 35-45. |
| 1. Muggeridge, DJ; Howe, CCF; Spendiff, O; Pedlar, C; James, PE; Easton, C (2014) A Single Dose of Beetroot Juice Enhances Cycling Performance in Simulated Altitude. MEDICINE AND SCIENCE IN SPORTS AND EXERCISE, 46:1, 143-150. |
| 1. Jones, AM (2014) Influence of dietary nitrate on the physiological determinants of exercise performance: a critical review. APPLIED PHYSIOLOGY NUTRITION AND METABOLISM, 39:9, 1019-1028. |
| 1. Hoon, MW; Hopkins, WG; Jones, AM; Martin, DT; Halson, SL; West, NP; Johnson, NA; Burke, LM (2014) Nitrate supplementation and high-intensity performance in competitive cyclists. APPLIED PHYSIOLOGY NUTRITION AND METABOLISM-PHYSIOLOGIE APPLIQUEE NUTRITION ET METABOLISME, 39:9, 1043-1049. |
| 1. Hoon, MW; Jones, AM; Johnson, NA; Blackwell, JR; Broad, EM; Lundy, B; Rice, AJ; Burke, LM (2014) The Effect of Variable Doses of Inorganic Nitrate-Rich Beetroot Juice on Simulated 2000-m Rowing Performance in Trained Athletes. INTERNATIONAL JOURNAL OF SPORTS PHYSIOLOGY AND PERFORMANCE, 9:4, 615-620. |
| 1. Berry, MJ; Justus, NW; Hauser, JI; Case, AH; Helms, CC; Basu, S; Rogers, Z; Lewis, MT; Miller, GD (2015) Dietary nitrate supplementation improves exercise performance and decreases blood pressure in COPD patients. NITRIC OXIDE-BIOLOGY AND CHEMISTRY, 48:, 22-30. |
| 1. Affourtit, C; Bailey, SJ; Jones, AM; Smallwood, MJ; Winyard, PG (2015) On the mechanism by which dietary nitrate improves human skeletal muscle function. FRONTIERS IN PHYSIOLOGY, 6:, -. |
| 1. Curtis, KJ; O'Brien, KA; Tanner, RJ; Polkey, JI; Minnion, M; Feelisch, M; Polkey, MI; Edwards, LM; Hopkinson, NS (2015) Acute Dietary Nitrate Supplementation and Exercise Performance in COPD: A Double-Blind, Placebo-Controlled, Randomised Controlled Pilot Study. PLOS ONE, 10:12, -. |
| 1. Poortmans, JR; Gualano, B; Carpentier, A (2015) Nitrate supplementation and human exercise performance: too much of a good thing?. CURRENT OPINION IN CLINICAL NUTRITION AND METABOLIC CARE, 18:6, 599-604. |
| 1. Flueck, JL; Bogdanova, A; Mettler, S; Perret, C (2016) Is beetroot juice more effective than sodium nitrate? The effects of equimolar nitrate dosages of nitrate-rich beetroot juice and sodium nitrate on oxygen consumption during exercise. APPLIED PHYSIOLOGY NUTRITION AND METABOLISM, 41:4, 421-429. |
| 1. Siervo, M; Oggioni, C; Jakovljevic, DG; Trenell, M; Mathers, JC; Houghton, D; Celis-Morales, C; Ashor, AW; Ruddock, A; Ranchordas, M; Klonizakis, M; Williams, EA (2016) Dietary nitrate does not affect physical activity or outcomes in healthy older adults in a randomized, cross-over trial. NUTRITION RESEARCH, 36:12, 1361-1369. |
| 1. Vasconcellos, J; Silvestre, DH; Baiao, DD; Werneck-de-Castro, JP; Alvares, TS; Paschoalin, VMF (2017) A Single Dose of Beetroot Gel Rich in Nitrate Does Not Improve Performance but Lowers Blood Glucose in Physically Active Individuals. JOURNAL OF NUTRITION AND METABOLISM, :, -. |
| 1. Nyakayiru, J; Jonvik, KL; Trommelen, J; Pinckaers, PJM; Senden, JM; van Loon, LJC; Verdijk, LB (2017) Beetroot Juice Supplementation Improves High-Intensity Intermittent Type Exercise Performance in Trained Soccer Players. NUTRIENTS, 9:3, -. |
| 1. Lowings, S; Shannon, OM; Deighton, K; Matu, J; Barlow, MJ (2017) Effect of Dietary Nitrate Supplementation on Swimming Performance in Trained Swimmers. INTERNATIONAL JOURNAL OF SPORT NUTRITION AND EXERCISE METABOLISM, 27:4, 377-384. |
| 1. Buhl, KR; Rodrigues, L (2017) Dietary nitrate supplementation in running physical performance. RBNE-REVISTA BRASILEIRA DE NUTRICAO ESPORTIVA, 11:63, 353-362. |
| 1. Dominguez, R; Mate-Munoz, JL; Cuenca, E; Garcia-Fernandez, P; Mata-Ordonez, F; Lozano-Estevan, MC; Veiga-Herreros, P; da Silva, SF; Garnacho-Castano, MV (2018) Effects of beetroot juice supplementation on intermittent high-intensity exercise efforts. JOURNAL OF THE INTERNATIONAL SOCIETY OF SPORTS NUTRITION, 15:, -. |
| 1. Richards, JC; Racine, ML; Hearon, CM; Kunkel, M; Luckasen, GJ; Larson, DG; Allen, JD; Dinenno, FA (2018) Acute ingestion of dietary nitrate increases muscle blood flow via local vasodilation during handgrip exercise in young adults. PHYSIOLOGICAL REPORTS, 6:2, |
| 1. Jonvik, KL; Nyakayiru, J; Van Dijk, JW; Maase, K; Ballak, SB; Senden, JMG; Van Loon, LJC; Verdijk, LB (2018) Repeated-sprint performance and plasma responses following beetroot juice supplementation do not differ between recreational, competitive and elite sprint athletes. EUROPEAN JOURNAL OF SPORT SCIENCE, 18:4, 524-533. |
| 1. Oskarsson, J; McGawley, K (2018) No individual or combined effects of caffeine and beetroot-juice supplementation during submaximal or maximal running. APPLIED PHYSIOLOGY NUTRITION AND METABOLISM, 43:7, 697-703. |
| 1. Jones, AM; Thompson, C; Wylie, LJ; Vanhatalo, A (2018) Dietary Nitrate and Physical Performance. ANNUAL REVIEW OF NUTRITION, VOL 38, 38:, 303-328. |
| 1. Kerley, CP; James, PE; McGowan, A; Faul, J; Cormican, L (2019) Dietary nitrate improved exercise capacity in COPD but not blood pressure or pulmonary function: a 2 week, double-blind randomised, placebo-controlled crossover trial. INTERNATIONAL JOURNAL OF FOOD SCIENCES AND NUTRITION, 70:2, 222-231. |

**13) Oxidative stress and dietary supplement use**


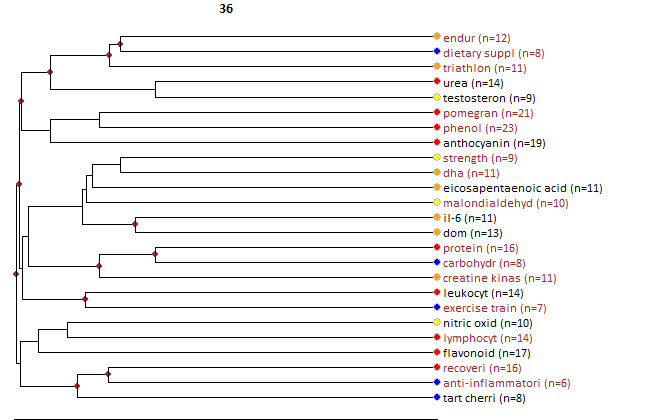


Core documents

| 1. Fischer, CP; Hiscock, NJ; Penkowa, M; Basu, S; Vessby, B; Kallner, A; Sjoberg, LB; Pedersen, BK (2004) Supplementation with vitamins C and E inhibits the release of interleukin-6 from contracting human skeletal muscle. JOURNAL OF PHYSIOLOGY-LONDON, 558:2, 633-645. |
| --- |
| 1. Morillas-Ruiz, JM; Garcia, JAV; Lopez, FJ; Vidal-Guevara, ML; Zafrilla, P (2006) Effects of polyphenolic antioxidants on exercise-induced oxidative stress. CLINICAL NUTRITION, 25:3, 444-453. |
| 1. Skarpanska-Stejnborn, A; Pilaczynska-Szczesniak, L; Basta, P; Deskur-Smielecka, E; Horoszkiewicz-Hassan, M (2008) The influence of supplementation with artichoke (Cynara scolymus L.) extract on selected redox parameters in rowers. INTERNATIONAL JOURNAL OF SPORT NUTRITION AND EXERCISE METABOLISM, 18:3, 313-327. |
| 1. Sureda, A; Tauler, P; Aguilo, A; Cases, N; Llompart, I; Tur, JA; Pons, A (2008) Influence of an antioxidant vitamin-enriched drink on pre- and post-exercise lymphocyte antioxidant system. ANNALS OF NUTRITION AND METABOLISM, 52:3, 233-240. |
| 1. Powers, SK; Jackson, MJ (2008) Exercise-induced oxidative stress: Cellular mechanisms and impact on muscle force production. PHYSIOLOGICAL REVIEWS, 88:4, 1243-1276. |
| 1. Bloomer, RJ; Larson, DE; Fisher-Wellman, KH; Galpin, AJ; Schilling, BK (2009) Effect of eicosapentaenoic and docosahexaenoic acid on resting and exercise-induced inflammatory and oxidative stress biomarkers: a randomized, placebo controlled, cross-over study. LIPIDS IN HEALTH AND DISEASE, 8:, -. |
| 1. Howatson, G; McHugh, MP; Hill, JA; Brouner, J; Jewell, AP; van Someren, KA; Shave, RE; Howatson, SA (2010) Influence of tart cherry juice on indices of recovery following marathon running. SCANDINAVIAN JOURNAL OF MEDICINE & SCIENCE IN SPORTS, 20:6, 843-852. |
| 1. Bailey, DM; Williams, C; Betts, JA; Thompson, D; Hurst, TL (2011) Oxidative stress, inflammation and recovery of muscle function after damaging exercise: effect of 6-week mixed antioxidant supplementation. EUROPEAN JOURNAL OF APPLIED PHYSIOLOGY, 111:6, 925-936. |
| 1. Jowko, E; Sacharuk, J; Balasinska, B; Ostaszewski, P; Charmas, M; Charmas, R (2011) Green tea extract supplementation gives protection against exercise-induced oxidative damage in healthy men. NUTRITION RESEARCH, 31:11, 813-821. |
| 1. McAnulty, LS; Nieman, DC; Dumke, CL; Shooter, LA; Henson, DA; Utter, AC; Milne, G; McAnulty, SR (2011) Effect of blueberry ingestion on natural killer cell counts, oxidative stress, and inflammation prior to and after 2.5 h of running. APPLIED PHYSIOLOGY NUTRITION AND METABOLISM-PHYSIOLOGIE APPLIQUEE NUTRITION ET METABOLISME, 36:6, 976-984. |
| 1. McLeay, Y; Barnes, MJ; Mundel, T; Hurst, SM; Hurst, RD; Stannard, SR (2012) Effect of New Zealand blueberry consumption on recovery from eccentric exercise-induced muscle damage. JOURNAL OF THE INTERNATIONAL SOCIETY OF SPORTS NUTRITION, 9:, -. |
| 1. Deminice, R; Rosa, FT; Franco, GS; Jordao, AA; de Freitas, EC (2013) Effects of creatine supplementation on oxidative stress and inflammatory markers after repeated-sprint exercise in humans. NUTRITION, 29:9, 1127-1132. |
| 1. Capo, X; Martorell, M; Sureda, A; Llompart, I; Tur, JA; Pons, A (2015) Diet supplementation with DHA-enriched food in football players during training season enhances the mitochondrial antioxidant capabilities in blood mononuclear cells. EUROPEAN JOURNAL OF NUTRITION, 54:1, 35-49. |
| 1. Ceci, R; Duranti, G; Sgro, P; Sansone, M; Guidetti, L; Baldari, C; Sabatini, S; Di Luigi, L (2015) Effects of tadalafil administration on plasma markers of exercise-induced muscle damage, IL6 and antioxidant status capacity. EUROPEAN JOURNAL OF APPLIED PHYSIOLOGY, 115:3, 531-539. |
| 1. Bell, PG; Walshe, IH; Davison, GW; Stevenson, EJ; Howatson, G (2015) Recovery facilitation with Montmorency cherries following high-intensity, metabolically challenging exercise. APPLIED PHYSIOLOGY NUTRITION AND METABOLISM, 40:4, -. |
| 1. McCormick, R; Peeling, P; Binnie, M; Dawson, B; Sim, M (2016) Effect of tart cherry juice on recovery and next day performance in well-trained Water Polo players. JOURNAL OF THE INTERNATIONAL SOCIETY OF SPORTS NUTRITION, 13:, -. |
| 1. Capo, X; Martorell, M; Busquets-Cortes, C; Sureda, A; Riera, J; Drobnic, F; Tur, JA; Pons, A (2016) Effects of dietary almond- and olive oil-based docosahexaenoic acid- and vitamin E-enriched beverage supplementation on athletic performance and oxidative stress markers. FOOD & FUNCTION, 7:12, 4920-4934. |
| 1. Peake, JM; Neubauer, O; Della Gatta, PA; Nosaka, K (2017) Muscle damage and inflammation during recovery from exercise. JOURNAL OF APPLIED PHYSIOLOGY, 122:3, 559-570. |
| 1. Urbaniak, A; Basta, P; Ast, K; Woloszyn, A; Kurianska-Woloszyn, J; Latour, E; Skarpanska-Stejnborn, A (2018) The impact of supplementation with pomegranate fruit (Punica granatum L.) juice on selected antioxidant parameters and markers of iron metabolism in rowers. JOURNAL OF THE INTERNATIONAL SOCIETY OF SPORTS NUTRITION, 15:, -. |
| 1. Peres, A; Dorneles, GP; Boeira, MCR; Schipper, LL; Beretta, A; Vilela, T; Andrade, VM; Romao, PRT (2018) Acute fish oil supplementation modulates the inflammatory response after strenuous exercise in obese men: A cross-over study. PROSTAGLANDINS LEUKOTRIENES AND ESSENTIAL FATTY ACIDS, 137:, 5-11. |

**14) Dietary supplement use and doping**


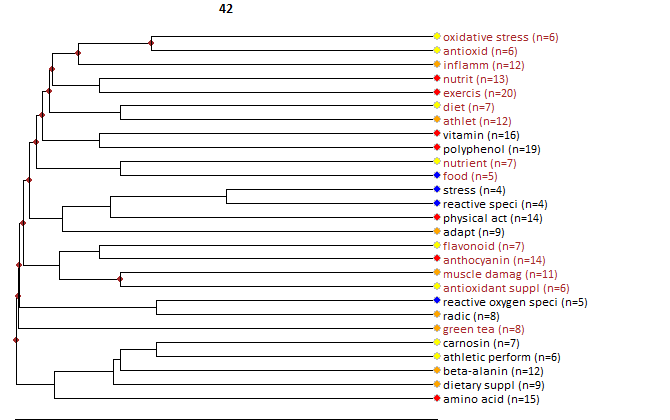


Core documents

| 1. Powers, SK; DeRuisseau, KC; Quindry, J; Hamilton, KL (2004) Dietary antioxidants and exercise. JOURNAL OF SPORTS SCIENCES, 22:1, 81-94. |
| --- |
| 1. McGinley, C; Shafat, A; Donnelly, AE (2009) Does Antioxidant Vitamin Supplementation Protect against Muscle Damage?. SPORTS MEDICINE, 39:12, 1011-1032. |
| 1. Peternelj, TT; Coombes, JS (2011) Antioxidant Supplementation during Exercise Training Beneficial or Detrimental?. SPORTS MEDICINE, 41:12, 1043-1069. |
| 1. Yiannakopoulou, EC (2013) Targeting oxidative stress response by green tea polyphenols: clinical implications. FREE RADICAL RESEARCH, 47:9, 667-671. |
| 1. Draeger, CL; Naves, A; Marques, N; Baptistella, AB; Carnauba, RA; Paschoal, V; Nicastro, H (2014) Controversies of antioxidant vitamins supplementation in exercise: ergogenic or ergolytic effects in humans?. JOURNAL OF THE INTERNATIONAL SOCIETY OF SPORTS NUTRITION, 11:, -. |
| 1. Sureda, A; Tejada, S; Bibiloni, MD; Tur, JA; Pons, A (2014) Polyphenols: Well Beyond The Antioxidant Capacity: Polyphenol Supplementation and Exercise-Induced Oxidative Stress and Inflammation. CURRENT PHARMACEUTICAL BIOTECHNOLOGY, 15:4, 373-379. |
| 1. Pingitore, A; Lima, GPP; Mastorci, F; Quinones, A; Iervasi, G; Vassalle, C (2015) Exercise and oxidative stress: Potential effects of antioxidant dietary strategies in sports. NUTRITION, 31:43654, 916-922. |
| 1. Kasote, DM; Katyare, SS; Hegde, MV; Bae, H (2015) Significance of Antioxidant Potential of Plants and its Relevance to Therapeutic Applications. INTERNATIONAL JOURNAL OF BIOLOGICAL SCIENCES, 11:8, 982-991. |
| 1. Boccatonda, A; Tripaldi, R; Davi, G; Santilli, F (2016) Oxidative Stress Modulation Through Habitual Physical Activity. CURRENT PHARMACEUTICAL DESIGN, 22:24, 3648-3680. |
| 1. Tejada, S; Nabavi, SM; Capo, X; Martorell, M; Bibiloni, MD; Tur, JA; Pons, A; Sureda, A (2017) Quercetin Effects on Exercise Induced Oxidative Stress and Inflammation. CURRENT ORGANIC CHEMISTRY, 21:4, 348-356. |
| 1. Lipinski, K; Mazur, M; Antoszkiewicz, Z; Purwin, C (2017) POLYPHENOLS IN MONOGASTRIC NUTRITION - A REVIEW. ANNALS OF ANIMAL SCIENCE, 17:1, 41-58. |

**15) Oxidative stress, inflammation and dietary antioxidants**


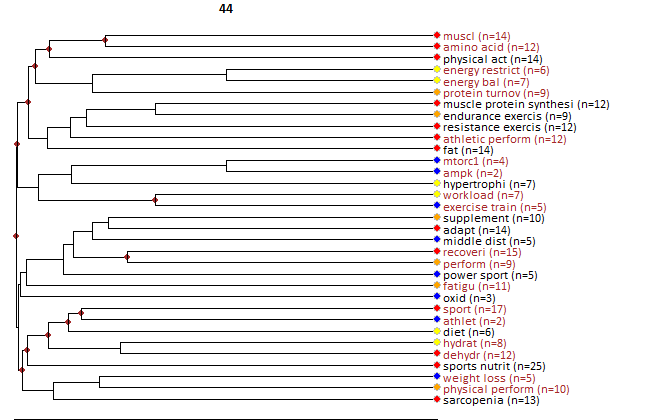


Core documents

| 1. Burke, LM; Kiens, B; Ivy, JL (2004) Carbohydrates and fat for training and recovery. JOURNAL OF SPORTS SCIENCES, 22:1, 15-30. |
| --- |
| 1. Campbell, B; Kreider, RB; Ziegenfuss, T; La Bounty, P; Roberts, M; Burke, D; Landis, J; Lopez, H; Antonio, J (2007) International Society of Sports Nutrition position stand: protein and exercise. JOURNAL OF THE INTERNATIONAL SOCIETY OF SPORTS NUTRITION, 4:, -. |
| 1. Beck, KL; Thomson, JS; Swift, RJ; von Hurst, PR (2015) Role of nutrition in performance enhancement and postexercise recovery. OPEN ACCESS JOURNAL OF SPORTS MEDICINE, 6:, 259-267. |
| 1. Coffey, VG; Hawley, JA (2007) The molecular bases of training adaptation. SPORTS MEDICINE, 37:9, 737-763. |
| 1. Broad, EM; Cox, GR (2008) What is the optimal composition of an athlete's diet?. EUROPEAN JOURNAL OF SPORT SCIENCE, 8:2, 57-65. |
| 1. Kreider, RB; Wilborn, CD; Taylor, L; Campbell, B; Almada, AL; Collins, R; Cooke, M; Earnest, CP; Greenwood, M; Kalman, DS; Kerksick, CM; Kleiner, SM; Leutholtz, B; Lopez, H; Lowery, LM; Mendel, R; Smith, A; Spano, M; Wildman, R; Willoughby, DS; Ziegenfuss, TN; Antonio, J (2010) ISSN exercise & sport nutrition review: research & recommendations. JOURNAL OF THE INTERNATIONAL SOCIETY OF SPORTS NUTRITION, 7:, -. |
| 1. Burke, LM (2010) Fueling strategies to optimize performance: training high or training low?. SCANDINAVIAN JOURNAL OF MEDICINE & SCIENCE IN SPORTS, 20:, 48-58. |
| 1. Hawley, JA; Burke, LM; Phillips, SM; Spriet, LL (2011) Nutritional modulation of training-induced skeletal muscle adaptations. JOURNAL OF APPLIED PHYSIOLOGY, 110:3, 834-845. |
| 1. Hausswirth, C; Le Meur, Y (2011) Physiological and Nutritional Aspects of Post-Exercise Recovery Specific Recommendations for Female Athletes. SPORTS MEDICINE, 41:10, 861-882. |
| 1. Stellingwerff, T; Maughan, RJ; Burke, LM (2011) Nutrition for power sports: Middle-distance running, track cycling, rowing, canoeing/kayaking, and swimming. JOURNAL OF SPORTS SCIENCES, 29:, S79-S89. |
| 1. Churchward-Venne, TA; Burd, NA; Phillips, SM (2012) Nutritional regulation of muscle protein synthesis with resistance exercise: strategies to enhance anabolism. NUTRITION & METABOLISM, 9:, -. |
| 1. Aragon, AA; Schoenfeld, BJ (2013) Nutrient timing revisited: is there a post-exercise anabolic window?. JOURNAL OF THE INTERNATIONAL SOCIETY OF SPORTS NUTRITION, 10:, -. |
| 1. Ross, M; Abbiss, C; Laursen, P; Martin, D; Burke, L (2013) Precooling Methods and Their Effects on Athletic Performance A Systematic Review and Practical Applications. SPORTS MEDICINE, 43:3, 207-225. |
| 1. Hawley, JA (2013) Nutritional Strategies to Modulate the Adaptive Response to Endurance Training. NUTRITIONAL COACHING STRATEGY TO MODULATE TRAINING EFFICIENCY, 75:, 1-14. |
| 1. van Loon, LJC; Tipton, KD (2013) Concluding Remarks: Nutritional Strategies to Support the Adaptive Response to Prolonged Exercise Training. NUTRITIONAL COACHING STRATEGY TO MODULATE TRAINING EFFICIENCY, 75:, 135-141. |
| 1. van Loon, LJC; Meeusen, R (2013) Concluding Remarks: Nutritional Strategies to Increase Performance Capacity. LIMITS OF HUMAN ENDURANCE, 76:, 121-125. |
| 1. McLellan, TM; Pasiakos, SM; Lieberman, HR (2014) Effects of Protein in Combination with Carbohydrate Supplements on Acute or Repeat Endurance Exercise Performance: A Systematic Review. SPORTS MEDICINE, 44:4, 535-550. |
| 1. Bellinger, PM (2014) beta-ALANINE SUPPLEMENTATION FOR ATHLETIC PERFORMANCE: AN UPDATE. JOURNAL OF STRENGTH AND CONDITIONING RESEARCH, 28:6, 1751-1770. |
| 1. Mujika, I; Stellingwerff, T; Tipton, K (2014) Nutrition and Training Adaptations in Aquatic Sports. INTERNATIONAL JOURNAL OF SPORT NUTRITION AND EXERCISE METABOLISM, 24:4, 414-424. |
| 1. Burke, LM; Mujika, I (2014) Nutrition for Recovery in Aquatic Sports. INTERNATIONAL JOURNAL OF SPORT NUTRITION AND EXERCISE METABOLISM, 24:4, 425-436. |
| 1. Russell, M; Kingsley, M (2014) The Efficacy of Acute Nutritional Interventions on Soccer Skill Performance. SPORTS MEDICINE, 44:7, 957-970. |
| 1. Kumstat, M (2013) A current literature review Actual concepts and contemporary scientific interests in sport nutrition. SPORT AND QUALITY OF LIFE 2013, :, 59-66. |
| 1. Burke, LM (2015) Re-Examining High-Fat Diets for Sports Performance: Did We Call the 'Nail in the Coffin' Too Soon?. SPORTS MEDICINE, 45:, S33-S49. |
| 1. Witard, OC; Wardle, SL; Macnaughton, LS; Hodgson, AB; Tipton, KD (2016) Protein Considerations for Optimising Skeletal Muscle Mass in Healthy Young and Older Adults. NUTRIENTS, 8:4, -. |
| 1. Phillips, SM; Chevalier, S; Leidy, HJ (2016) Protein "requirements" beyond the RDA: implications for optimizing health. APPLIED PHYSIOLOGY NUTRITION AND METABOLISM, 41:5, 565-+. |
| 1. Witard, OC; McGlory, C; Hamilton, DL; Phillips, SM (2016) Growing older with health and vitality: a nexus of physical activity, exercise and nutrition. BIOGERONTOLOGY, 17:3, 529-546. |
| 1. Close, GL; Hamilton, DL; Philp, A; Burke, LM; Morton, JP (2016) New strategies in sport nutrition to increase exercise performance. FREE RADICAL BIOLOGY AND MEDICINE, 98:, 144-158. |
| 1. Egan, B (2016) Protein intake for athletes and active adults: Current concepts and controversies. NUTRITION BULLETIN, 41:3, 202-213. |
| 1. Escobar, KA; VanDusseldorp, TA; Kerksick, CM (2016) Carbohydrate intake and resistance-based exercise: are current recommendations reflective of actual need?. BRITISH JOURNAL OF NUTRITION, 116:12, 2053-2065. |
| 1. Applegate, C; Mueller, M; Zuniga, KE (2017) Influence of Dietary Acid Load on Exercise Performance. INTERNATIONAL JOURNAL OF SPORT NUTRITION AND EXERCISE METABOLISM, 27:3, 213-219. |
| 1. Jeukendrup, AE (2017) Periodized Nutrition for Athletes. SPORTS MEDICINE, 47:, S51-S63. |
| 1. Burke, LM (2017) Practical Issues in Evidence-Based Use of Performance Supplements: Supplement Interactions, Repeated Use and Individual Responses. SPORTS MEDICINE, 47:, S79-S100. |
| 1. Burke, LM; Peeling, P (2018) Methodologies for Investigating Performance Changes With Supplement Use. INTERNATIONAL JOURNAL OF SPORT NUTRITION AND EXERCISE METABOLISM, 28:2, 159-169. |
| 1. Mujika, I; Halson, S; Burke, LM; Balague, G; Farrow, D (2018) An Integrated, Multifactorial Approach to Periodization for Optimal Performance in Individual and Team Sports. INTERNATIONAL JOURNAL OF SPORTS PHYSIOLOGY AND PERFORMANCE, 13:5, 538-561. |
| 1. Casazza, GA; Tovar, AP; Richardson, CE; Cortez, AN; Davis, BA (2018) Energy Availability, Macronutrient Intake, and Nutritional Supplementation for Improving Exercise Performance in Endurance Athletes. CURRENT SPORTS MEDICINE REPORTS, 17:6, 215-223. |
| 1. Kerksick, CM; Wilborn, CD; Roberts, MD; Smith-Ryan, A; Kleiner, SM; Jager, R; Collins, R; Cooke, M; Davis, JN; Galvan, E; Greenwood, M; Lowery, LM; Wildman, R; Antonio, J; Kreider, RB (2018) ISSN exercise & sports nutrition review update: research & recommendations. JOURNAL OF THE INTERNATIONAL SOCIETY OF SPORTS NUTRITION, 15:, -. |
| 1. Burke, LM; Hawley, JA; Jeukendrup, A; Morton, JP; Stellingwerff, T; Maughan, RJ (2018) Toward a Common Understanding of Diet-Exercise Strategies to Manipulate Fuel Availability for Training and Competition Preparation in Endurance Sport. INTERNATIONAL JOURNAL OF SPORT NUTRITION AND EXERCISE METABOLISM, 28:5, 451-463. |
| 1. Murphy, CH; Roche, HM (2018) Nutrition and physical activity countermeasures for sarcopenia: Time to get personal?. NUTRITION BULLETIN, 43:4, 374-387. |
| 1. Valenta, R; Dorofeeva, YA (2018) Sport nutrition: the role of macronutrients and minerals in endurance exercises. FOODS AND RAW MATERIALS, 6:2, 403-412. |

**16) Training adaptation and nutritional strategies**


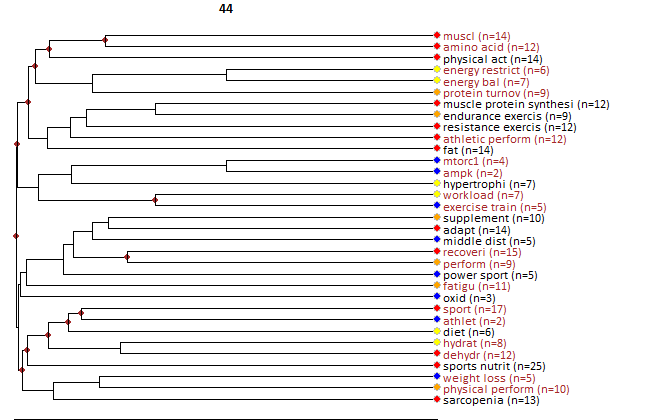


Core documents

| 1. Burke, LM; Kiens, B; Ivy, JL (2004) Carbohydrates and fat for training and recovery. JOURNAL OF SPORTS SCIENCES, 22:1, 15-30. |
| --- |
| 1. Campbell, B; Kreider, RB; Ziegenfuss, T; La Bounty, P; Roberts, M; Burke, D; Landis, J; Lopez, H; Antonio, J (2007) International Society of Sports Nutrition position stand: protein and exercise. JOURNAL OF THE INTERNATIONAL SOCIETY OF SPORTS NUTRITION, 4:, -. |
| 1. Beck, KL; Thomson, JS; Swift, RJ; von Hurst, PR (2015) Role of nutrition in performance enhancement and postexercise recovery. OPEN ACCESS JOURNAL OF SPORTS MEDICINE, 6:, 259-267. |
| 1. Coffey, VG; Hawley, JA (2007) The molecular bases of training adaptation. SPORTS MEDICINE, 37:9, 737-763. |
| 1. Broad, EM; Cox, GR (2008) What is the optimal composition of an athlete's diet?. EUROPEAN JOURNAL OF SPORT SCIENCE, 8:2, 57-65. |
| 1. Kreider, RB; Wilborn, CD; Taylor, L; Campbell, B; Almada, AL; Collins, R; Cooke, M; Earnest, CP; Greenwood, M; Kalman, DS; Kerksick, CM; Kleiner, SM; Leutholtz, B; Lopez, H; Lowery, LM; Mendel, R; Smith, A; Spano, M; Wildman, R; Willoughby, DS; Ziegenfuss, TN; Antonio, J (2010) ISSN exercise & sport nutrition review: research & recommendations. JOURNAL OF THE INTERNATIONAL SOCIETY OF SPORTS NUTRITION, 7:, -. |
| 1. Burke, LM (2010) Fueling strategies to optimize performance: training high or training low?. SCANDINAVIAN JOURNAL OF MEDICINE & SCIENCE IN SPORTS, 20:, 48-58. |
| 1. Hawley, JA; Burke, LM; Phillips, SM; Spriet, LL (2011) Nutritional modulation of training-induced skeletal muscle adaptations. JOURNAL OF APPLIED PHYSIOLOGY, 110:3, 834-845. |
| 1. Hausswirth, C; Le Meur, Y (2011) Physiological and Nutritional Aspects of Post-Exercise Recovery Specific Recommendations for Female Athletes. SPORTS MEDICINE, 41:10, 861-882. |
| 1. Stellingwerff, T; Maughan, RJ; Burke, LM (2011) Nutrition for power sports: Middle-distance running, track cycling, rowing, canoeing/kayaking, and swimming. JOURNAL OF SPORTS SCIENCES, 29:, S79-S89. |
| 1. Churchward-Venne, TA; Burd, NA; Phillips, SM (2012) Nutritional regulation of muscle protein synthesis with resistance exercise: strategies to enhance anabolism. NUTRITION & METABOLISM, 9:, -. |
| 1. Aragon, AA; Schoenfeld, BJ (2013) Nutrient timing revisited: is there a post-exercise anabolic window?. JOURNAL OF THE INTERNATIONAL SOCIETY OF SPORTS NUTRITION, 10:, -. |
| 1. Ross, M; Abbiss, C; Laursen, P; Martin, D; Burke, L (2013) Precooling Methods and Their Effects on Athletic Performance A Systematic Review and Practical Applications. SPORTS MEDICINE, 43:3, 207-225. |
| 1. Hawley, JA (2013) Nutritional Strategies to Modulate the Adaptive Response to Endurance Training. NUTRITIONAL COACHING STRATEGY TO MODULATE TRAINING EFFICIENCY, 75:, 1-14. |
| 1. van Loon, LJC; Tipton, KD (2013) Concluding Remarks: Nutritional Strategies to Support the Adaptive Response to Prolonged Exercise Training. NUTRITIONAL COACHING STRATEGY TO MODULATE TRAINING EFFICIENCY, 75:, 135-141. |
| 1. van Loon, LJC; Meeusen, R (2013) Concluding Remarks: Nutritional Strategies to Increase Performance Capacity. LIMITS OF HUMAN ENDURANCE, 76:, 121-125. |
| 1. McLellan, TM; Pasiakos, SM; Lieberman, HR (2014) Effects of Protein in Combination with Carbohydrate Supplements on Acute or Repeat Endurance Exercise Performance: A Systematic Review. SPORTS MEDICINE, 44:4, 535-550. |
| 1. Bellinger, PM (2014) beta-ALANINE SUPPLEMENTATION FOR ATHLETIC PERFORMANCE: AN UPDATE. JOURNAL OF STRENGTH AND CONDITIONING RESEARCH, 28:6, 1751-1770. |
| 1. Mujika, I; Stellingwerff, T; Tipton, K (2014) Nutrition and Training Adaptations in Aquatic Sports. INTERNATIONAL JOURNAL OF SPORT NUTRITION AND EXERCISE METABOLISM, 24:4, 414-424. |
| 1. Burke, LM; Mujika, I (2014) Nutrition for Recovery in Aquatic Sports. INTERNATIONAL JOURNAL OF SPORT NUTRITION AND EXERCISE METABOLISM, 24:4, 425-436. |
| 1. Russell, M; Kingsley, M (2014) The Efficacy of Acute Nutritional Interventions on Soccer Skill Performance. SPORTS MEDICINE, 44:7, 957-970. |
| 1. Kumstat, M (2013) A current literature review Actual concepts and contemporary scientific interests in sport nutrition. SPORT AND QUALITY OF LIFE 2013, :, 59-66. |
| 1. Burke, LM (2015) Re-Examining High-Fat Diets for Sports Performance: Did We Call the 'Nail in the Coffin' Too Soon?. SPORTS MEDICINE, 45:, S33-S49. |
| 1. Witard, OC; Wardle, SL; Macnaughton, LS; Hodgson, AB; Tipton, KD (2016) Protein Considerations for Optimising Skeletal Muscle Mass in Healthy Young and Older Adults. NUTRIENTS, 8:4, -. |
| 1. Phillips, SM; Chevalier, S; Leidy, HJ (2016) Protein "requirements" beyond the RDA: implications for optimizing health. APPLIED PHYSIOLOGY NUTRITION AND METABOLISM, 41:5, 565-+. |
| 1. Witard, OC; McGlory, C; Hamilton, DL; Phillips, SM (2016) Growing older with health and vitality: a nexus of physical activity, exercise and nutrition. BIOGERONTOLOGY, 17:3, 529-546. |
| 1. Close, GL; Hamilton, DL; Philp, A; Burke, LM; Morton, JP (2016) New strategies in sport nutrition to increase exercise performance. FREE RADICAL BIOLOGY AND MEDICINE, 98:, 144-158. |
| 1. Egan, B (2016) Protein intake for athletes and active adults: Current concepts and controversies. NUTRITION BULLETIN, 41:3, 202-213. |
| 1. Escobar, KA; VanDusseldorp, TA; Kerksick, CM (2016) Carbohydrate intake and resistance-based exercise: are current recommendations reflective of actual need?. BRITISH JOURNAL OF NUTRITION, 116:12, 2053-2065. |
| 1. Applegate, C; Mueller, M; Zuniga, KE (2017) Influence of Dietary Acid Load on Exercise Performance. INTERNATIONAL JOURNAL OF SPORT NUTRITION AND EXERCISE METABOLISM, 27:3, 213-219. |
| 1. Jeukendrup, AE (2017) Periodized Nutrition for Athletes. SPORTS MEDICINE, 47:, S51-S63. |
| 1. Burke, LM (2017) Practical Issues in Evidence-Based Use of Performance Supplements: Supplement Interactions, Repeated Use and Individual Responses. SPORTS MEDICINE, 47:, S79-S100. |
| 1. Burke, LM; Peeling, P (2018) Methodologies for Investigating Performance Changes With Supplement Use. INTERNATIONAL JOURNAL OF SPORT NUTRITION AND EXERCISE METABOLISM, 28:2, 159-169. |
| 1. Mujika, I; Halson, S; Burke, LM; Balague, G; Farrow, D (2018) An Integrated, Multifactorial Approach to Periodization for Optimal Performance in Individual and Team Sports. INTERNATIONAL JOURNAL OF SPORTS PHYSIOLOGY AND PERFORMANCE, 13:5, 538-561. |
| 1. Casazza, GA; Tovar, AP; Richardson, CE; Cortez, AN; Davis, BA (2018) Energy Availability, Macronutrient Intake, and Nutritional Supplementation for Improving Exercise Performance in Endurance Athletes. CURRENT SPORTS MEDICINE REPORTS, 17:6, 215-223. |
| 1. Kerksick, CM; Wilborn, CD; Roberts, MD; Smith-Ryan, A; Kleiner, SM; Jager, R; Collins, R; Cooke, M; Davis, JN; Galvan, E; Greenwood, M; Lowery, LM; Wildman, R; Antonio, J; Kreider, RB (2018) ISSN exercise & sports nutrition review update: research & recommendations. JOURNAL OF THE INTERNATIONAL SOCIETY OF SPORTS NUTRITION, 15:, -. |
| 1. Burke, LM; Hawley, JA; Jeukendrup, A; Morton, JP; Stellingwerff, T; Maughan, RJ (2018) Toward a Common Understanding of Diet-Exercise Strategies to Manipulate Fuel Availability for Training and Competition Preparation in Endurance Sport. INTERNATIONAL JOURNAL OF SPORT NUTRITION AND EXERCISE METABOLISM, 28:5, 451-463. |
| 1. Murphy, CH; Roche, HM (2018) Nutrition and physical activity countermeasures for sarcopenia: Time to get personal?. NUTRITION BULLETIN, 43:4, 374-387. |
| 1. Valenta, R; Dorofeeva, YA (2018) Sport nutrition: the role of macronutrients and minerals in endurance exercises. FOODS AND RAW MATERIALS, 6:2, 403-412. |

**17) Gut microbiota**


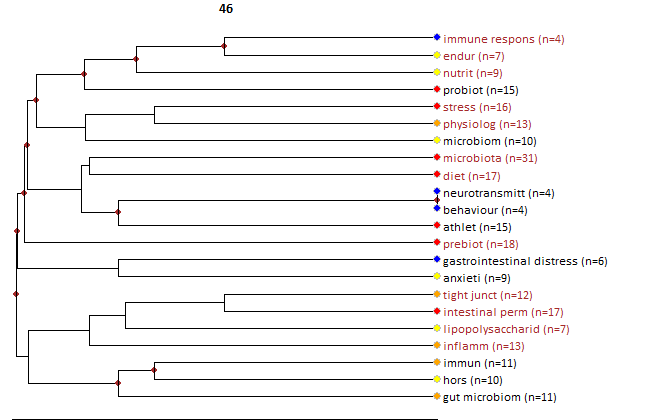


Core documents

| 1. Cresci, GA; Bawden, E (2015) Gut Microbiome: What We Do and Don't Know. NUTRITION IN CLINICAL PRACTICE, 30:6, 734-746. |
| --- |
| 1. Yuan, TF; Rocha, NBF; Paes, F; Arias-Carrion, O; Machado, S; de Sa, AS (2015) Neural Mechanisms of Exercise: Effects on Gut Miccrobiota and Depression. CNS & NEUROLOGICAL DISORDERS-DRUG TARGETS, 14:10, 1312-1314. |
| 1. Cronin, O; Molloy, MG; Shanahan, F (2016) Exercise, fitness, and the gut. CURRENT OPINION IN GASTROENTEROLOGY, 32:2, 67-73. |
| 1. Marchesi, JR; Adams, DH; Fava, F; Hermes, GDA; Hirschfield, GM; Hold, G; Quraishi, MN; Kinross, J; Smidt, H; Tuohy, KM; Thomas, LV; Zoetendal, EG; Hart, A (2016) The gut microbiota and host health: a new clinical frontier. GUT, 65:2, 330-339. |
| 1. Clark, A; Mach, N (2016) Exercise-induced stress behavior, gut-microbiota-brain axis and diet: a systematic review for athletes. JOURNAL OF THE INTERNATIONAL SOCIETY OF SPORTS NUTRITION, 13:, -. |
| 1. Volpe, SL (2017) The Gut Microbiota and Exercise Performance. ACSMS HEALTH & FITNESS JOURNAL, 21:3, 34-36. |
| 1. Clark, A; Mach, N (2017) The Crosstalk between the Gut Microbiota and Mitochondria during Exercise. FRONTIERS IN PHYSIOLOGY, 8:, -. |
| 1. Mach, N; Fuster-Botella, D (2017) Endurance exercise and gut microbiota: A review. JOURNAL OF SPORT AND HEALTH SCIENCE, 6:2, 179-197. |
| 1. Liu, ZH; Liu, HY; Zhou, HB; Zhan, Q; Lai, WY; Zeng, QC; Ren, H; Xu, DL (2017) Moderate-Intensity Exercise Affects Gut Microbiome Composition and Influences Cardiac Function in Myocardial Infarction Mice. FRONTIERS IN MICROBIOLOGY, 8:, -. |
| 1. Lach, G; Schellekens, H; Dinan, TG; Cryan, JF (2018) Anxiety, Depression, and the Microbiome: A Role for Gut Peptides. NEUROTHERAPEUTICS, 15:1, 36-59. |
| 1. Zhang, N; Ju, ZJ; Zuo, T (2018) Time for food: The impact of diet on gut microbiota and human health. NUTRITION, 51-52:, 80-85. |
| 1. Gonzalez-Gonzalez, M; Diaz-Zepeda, C; Eyzaguirre-Velasquez, J; Gonzalez-Arancibia, C; Bravo, JA; Julio-Pieper, M (2019) Investigating Gut Permeability in Animal Models of Disease. FRONTIERS IN PHYSIOLOGY, 9:, -. |
| 1. Tengeler, AC; Kozicz, T; Kiliaan, AJ (2018) Relationship between diet, the gut microbiota, and brain function. NUTRITION REVIEWS, 76:8, 603-617. |
| 1. Diether, NE; Willing, BP (2019) Microbial Fermentation of Dietary Protein: An Important Factor in Diet-Microbe-Host Interaction. MICROORGANISMS, 7:1, -. |

**18) Celiac disease**


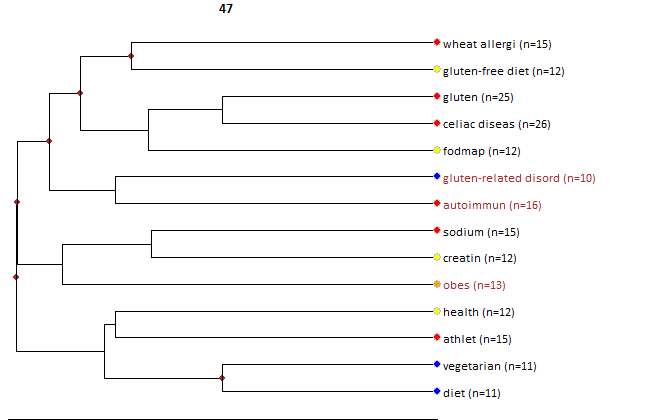


Core documents

| 1. Lebwohl, B; Ludvigsson, JF; Green, PHR (2015) Celiac disease and non-celiac gluten sensitivity. BMJ-BRITISH MEDICAL JOURNAL, 351:, -. |
| --- |
| 1. Krigel, A; Lebwohl, B (2016) Nonceliac Gluten Sensitivity. ADVANCES IN NUTRITION, 7:6, 1105-1110. |
| 1. Dittfeld, A; Gwizdek, K; Parol, D; Michalski, M (2018) Glutenfree diet: Characteristics of target groups. POSTEPY HIGIENY I MEDYCYNY DOSWIADCZALNEJ, 72:, -. |
