# Supplementary material for: Structure and trends of international sport nutrition research between 2000 and 2018: bibliometric mapping of sport nutrition science
Source: J Int Soc Sports Nutr. 2021 Feb 5;18:12. doi: 10.1186/s12970-021-00409-5 (PMC7866438; doi:10.1186/s12970-021-00409-5)
Supplement: Supplementary file 2 — Additional file 2. [file 12970_2021_409_MOESM2_ESM.docx]

In this additional file we describe the results of identifying so-called structural breakpoints in the time dynamics or growth of publication volume within our sample. We have applied two approaches: one test, based on F statistics, indicated one structural break at 2012. A further test, based on Bayesian Information Criterion yielded two breakpoints, one in 2003 and one in 2012. This also confirms that, given the low publication volume up to 2003, our analysis results reflect the period beginning with the 2000’s (Fig. 1).


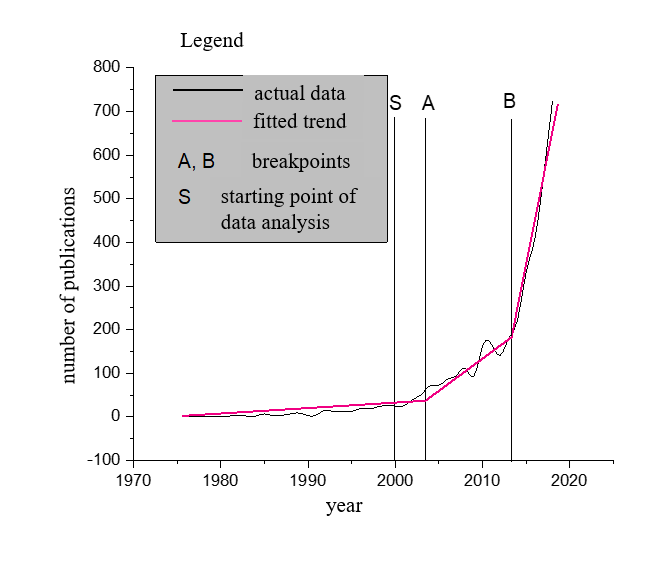


**Fig. 1** Structural breakpoints in growth of publication
